# The impact of supplementing quebracho and chestnut tannin extracts on the growth performance in growing and finishing beef cattle: a meta-analytical assessment

**DOI:** 10.1093/jas/skag082

**Published:** 2026-05-11

**Authors:** Mingyung Lee, Luis O Tedeschi

**Affiliations:** Department of Animal Science, Texas A&M University, College Station, TX 77843, USA; Department of Animal Science, Texas A&M University, College Station, TX 77843, USA

**Keywords:** meta-analysis, meta-regression, growing and finishing beef cattle, tannin extract, growth performance

## Abstract

Tannin extracts (TE), composed of condensed tannins from quebracho and hydrolyzable tannins from chestnut, have been studied as functional feed additives to improve growth performance in beef cattle, but reported effects vary with TE composition, dose, dietary protein, and co-additive. This meta-analysis evaluated TE supplementation effects on average daily gain (ADG), dry matter intake (DMI), and gain:feed (G:F) in growing and finishing beef cattle, using 162 treatment records from 43 published studies involving > 22,000 animals, mostly bulls and feedlot-based studies, including grazing and individually pen trials. Additional analyses included conditional independence tests for monensin use, forest and mosaic plots, and subset analyses for diets with or without monensin. Meta-regression indicated that TE supplementation was associated with increases in ADG (*P *< 0.001), DMI (*P *< 0.001), and G:F (*P *= 0.024). The ADG response was nonlinear; quadratic models indicated an optimal inclusion of ∼0.30% of dietary DM for maximizing weight gain. The disproportionate increase in ADG relative to DMI is consistent with improved nutrient utilization. The TE–ADG relationship was stronger at lower crude protein intake (*P *= 0.0525), however, because crude protein intake is not a direct proxy for rumen-degradable nitrogen supply and may co-vary with diet formulation and co-additives (e.g., ionophores and/or NPN sources), this pattern should be interpreted as context-dependent rather than as evidence of a universal mechanism. A conditional independence test also suggested that the TE-associated ADG responses were not independent of monensin use, suggesting a possible synergy effect, though additional studies are needed to confirm this pattern. Overall, TE appears to be a context-dependent additive rather than a universal growth promoter, and responses did not necessarily require doses near the model-derived optimum; benefits were also detectable at lower inclusions (e.g., ∼0.08% of dietary DM) in some contexts. Further research is needed to refine dosing strategies, better characterize TE chemistry, and evaluate environmental outcomes in dedicated studies.

## Teaser

Supplementing beef cattle diets with tannin extracts improved weight gain in a nonlinear, dose-dependent manner, with an optimal inclusion level around 0.30% of dietary dry matter and measurable benefits at approximately 0.08% under supportive conditions such as monensin-supplemented, lower-protein diets. These results show that tannins are not universal growth promoters but, when used at the right dose and in the right context, can improve efficiency of nutrient utilization and may help reduce environmental impacts.

## Introduction

Condensed (CT) and hydrolyzable (HT) tannins are plant-derived polyphenolic compounds commonly recognized as defensive chemicals, anti-nutritional factors, secondary metabolites, or phytochemicals (Tedeschi and Fox 2020). The CT consists of flavan-3-ol polymers, while HT are esters of gallic or ellagic acid bound to a polyol core, typically glucose (Salminen and Karonen 2011; Salminen et al. 2011). Both types can bind dietary proteins and, to a lesser extent, carbohydrates and minerals. In general, CT are reported to form pH-sensitive complexes with proteins that can influence ruminal protein degradation, whereas HT (gallo-/ellagitannins) have been reported to differ in binding mode and reactivity, with potential downstream effects on rumen microbiology and nitrogen utilization (Yamauchi et al. 2023; Yoshimura et al. 2025).

In beef cattle nutrition, CT and HT can have both beneficial and adverse effects depending on dose and production context. At moderate supplementation levels in the diet, both types can modulate ruminal protein metabolism and shift nitrogen use toward intestinal absorption, which may improve amino acid utilization and nitrogen-use efficiency (Tedeschi et al. 2014). From a practical standpoint, these mechanisms are particularly relevant in feedlot production, where cattle are intensively managed and dietary interventions are routinely used to improve growth efficiency and reduce nutrient losses. In this setting, improving nitrogen-use efficiency may also reduce nitrogen excretion and odor from manure (Hristov et al. 2011; Aboagye et al. 2018; Koenig et al. 2018). Tannins have also been reported to confer additional benefits in some contexts, including reducing enteric methane emissions and suppressing gastrointestinal parasites (Naumann et al. 2015; Naumann et al. 2017; Berça et al. 2023). However, at high dietary concentrations, they can inhibit microbial fermentation, decrease fiber digestibility, and reduce feed intake. These contrasting effects have motivated efforts to identify supplementation strategies that capture potential benefits while avoiding intake or digestibility penalties, thereby increasing interest in tannins as functional additives and in commercial tannin extracts (TE) designed for practical use in feeding programs (Tedeschi et al. 2014).

Previous studies have often examined CT and HT separately to assess their specific effects. However, most commercial TE products used in beef cattle diets are blends of both, typically combining quebracho (mainly CT) and chestnut (mostly HT) (Mueller‐Harvey 2006; Montano et al. 2022). Because the CT: HT ratio and analytical reporting can vary across products and studies, reported outcomes remain difficult to compare directly and may contribute to inconsistent results. This uncertainty continues to limit clear guidance for practical use of TE in cattle nutrition and parasite control (Tedeschi and Fox 2020). As a result, recent research has increasingly focused on evaluating the effects of TE blends on cattle growth performance.

Despite decades of research [e.g., McLeod (1974); McAllan (1992); Mueller-Harvey (2001); Min et al. (2003); Naumann et al. (2013)], findings on TE effects in beef cattle remain inconsistent. For example, supplementation with a chestnut–quebracho tannin blend (1.5 g/kg of dry matter; DM) in a high-grain diet increased final body weight, average daily gain (ADG), and dry matter intake (DMI) compared with monensin alone in finishing bulls (Nascimento et al. 2024), while HT from chestnut, alone or combined with CT from quebracho, reduced ruminal ammonia concentration without affecting growth performance in steers fed high-forage diets (Aboagye et al. 2018). In contrast, feeding quebracho tannins at 1%–2% of dietary DM in forage-based diets did not affect ADG or DMI and decreased crude protein digestibility (Beauchemin et al. 2007), and quebracho CT at 0.5%–1.0% of dietary DM in a high-grain finishing diet did not improve growth or gain:feed (G:F) (Ebert et al. 2017). These inconsistencies may stem from differences in TE source, dosage, diet composition, cattle breed, production phase, and other additives such as ionophores, antibiotics, or implants. Moreover, the available evidence is not evenly distributed across production settings. Many trials were conducted in feedlot systems, whereas grazing and individual-pen studies represent a smaller portion of the literature. As a result, our understanding of TE efficacy in beef cattle remains incomplete.

This study compiled a comprehensive database of published research on TE supplementation in growing and finishing beef cattle to address these uncertainties. Our objectives were to quantify the association of TE supplementation with ADG, DMI, and G:F and to evaluate whether response magnitude varies with TE dose and key study-level conditions captured in the dataset, including crude protein intake and common co-additives such as monensin. We hypothesized that TE supplementation would be associated with improved growth performance on average, but that responses would be heterogeneous and best explained by dose- and context-dependent moderation rather than a universal effect.

## Materials and methods

The meta-analytical approach adhered to the conceptual and statistical framework described by Tedeschi and Gorocica-Buenfil (2018), encompassing meta-regression, broken-line regression, and nonparametric evaluations. Where appropriate, methodological refinements and deviations from the original implementation were introduced and are explicitly described below.

### Dataset construction

The dataset used in this study was constructed to be comprehensive and to minimize publication bias by including both peer-reviewed articles and unpublished internal technical reports. A systematic search was conducted using Scopus and Google Scholar. Scopus was selected for its broad coverage of agricultural sciences and because it indexes the full content of ScienceDirect. Google Scholar was utilized to identify theses, dissertations, and conference proceedings that are often not indexed in databases like Web of Science. The search was performed using the following keyword combinations: (“tannin” OR “hydrolyzable tannin” OR “condensed tannin”) AND (“cattle” OR “bull” OR “steer” OR “heifer”) AND (“dry matter intake” OR “average daily gain” OR “feed efficiency”). To capture data from commercial production settings, where results often remain in internal reports rather than peer-reviewed journals, we deliberately included reports provided by tannin additive manufacturers. These sources were subject to the same strict screening and eligibility criteria as peer-reviewed literature to ensure data quality. To be included, a study was required to: 1) involve growing or finishing beef cattle; 2) use a defined source of TE; 3) include a control group (0% of dietary DM TE); 4) report quantitative data for ADG, DMI, or G:F with measures of variance (SE or SD); and 5) explicitly state the TE inclusion level on a dietary DM basis. Internal reports or studies that failed to meet these criteria or lacked sufficient methodological transparency were excluded.

The final dataset comprised 162 treatments extracted from 43 documents, all of which reported in vivo experiments (Table 1). Of the 43 sources, 6 were internal reports (PubID: 11, 32, 34, 35, 41, 42), 3 were theses (17, 21, 22), and the remainder were journal articles (e.g., 4, 5, 13, 15, 16, 18, 19, 23–28, 30, 31, 33, 36, 37) or conference proceedings (1–3, 6–10, 12, 14, 29, 38–40, 43). Most studies used a combination of CT and HT, but the CT: HT composition was rarely reported. Therefore, all values were standardized to a TE basis. When TE was not explicitly reported, it was calculated as the sum of CT and HT concentrations (reported in one study); when only TE was reported, composition was approximated as 70% CT and 30% HT (Montano et al. 2022). This approximation was used only as an imputation to harmonize reporting to a common TE scale and does not imply chemical or functional equivalence among commercial formulations.

**Table 1:** Characteristics of the in vivo studies included in the meta-analysis database and key descriptors.

					Feed additives (doses, % of dietary DM)^5^	Variables^6^					
PubID^1^	Sex^2^	Breed^3^	Type	Stage^4^		N	DOF	iBW	fBW	ADG	DMI
**1**	BU	BR	Beef	GR-FI	TE (0 to 0.32) + MN	40	226	184	511	1.45	8.7
**2**	BU	Mix	Beef	GR	TE (0 to 0.34) + MN + Urea	60	84	184	306	1.45	8
**3**	BU	Mix	Beef	FI	TE (0 to 0.32) + MN	60	98	366	506	1.43	9.4
**4**	BU	IN	Beef	—	TE (0 to 0.40) + MN	4	21	—	—	—	7.4
**5**	BU	NE	Beef	FI	TE (0 to 0.40)	27	136	318	486	1.23	9.1
**6**	BU	—	Beef	GR	TE (0 to 0.33) + MN	30	84	365	506	1.67	10.6
**7**	BU	—	Beef	—	TE (0 to 0.28) + MN	80	56	341	457	2.06	11.8
**8**	BU	—	Beef	—	TE (0 to 0.30) + MN + ZIL	80	108	342	529	1.75	11.7
**9**	HE	BR	Beef	GR	TE (0 to 0.28) + MN	40	84	196	276	0.95	6.3
**10**	BU	—	Beef	FI	TE (0 to 0.30) + MN + Cr	850	92	339	470	1.41	7.9
**11**	ST	BI	Beef	—	TE (0 to 0.37) + MN	225	62	224	312	1.41	8.1
**12**	BU	BR	Beef	—	TE (0 to 0.30) + MN + TMP	80	56	400	480	1.42	8.8
**13**	BU	NE	Beef	FI	TE (0 to 0.19) + MN + Urea	35	112	245	365	1.08	6.5
**14**	HE	AN	Beef	GR	TE (0 to 0.30) + MN	348	80	207	311	1.31	8.2
**15**	ST	HO	Beef	FI	TE (0 to 0.60) + MN + TY	96	84	392	520	1.53	10
**16**	ST	AN	Beef	FI	TE (0 to 1.00) + MN	27	126	336	601	2.1	11.4
**17**	BU	Mix	Beef	GR	TE (0 to 0.40) + MN	35	103	248	364	1.37	7.9
**18**	HE	—	Beef	—	TE (0 to 4.00)	5	23	—	—	—	—
**19**	ST	CO×BI	Beef	FI	TE (0 to 0.66) + MN + TY	144	154	349	599	1.64	9.9
**20**	ST	—	Beef	GR	TE (0 to 1.50)	75	84	293	354	0.73	7.6
**21**	ST	—	Beef	FI	TE (0 to 0.12) + MN + VM	180	150	359	576	1.44	11
**22**	BU	NE	Beef	FI	TE (0 to 0.21) + VM, YT	40	102	415	534	1.31	10
**23**	BU	NE	Beef	—	TE (0 to 0.10) + Urea	4	20	—	—	—	8.6
**24**	ST	BI	Beef	—	TE (0 to 4.50)	8	14	—	—	—	—
**25**	Mix	AN	Beef	GR	TE (0 to 0.23) + DFM	288	42	249	300	1.23	6.4
**26**	BU	NE	Beef	FI	TE (0 to 0.08) + MN	96	98	351	538	1.92	11
**27**	ST	NE	Beef	—	TE (0 to 0.10) + Urea	8	28	306	338	0.29	6.1
**28**	ST	HO	Dairy	GR	TE (0 to 0.40)	150	112	120	282	1.45	4.8
**29**	ST	HO	Dairy	GR/FI	TE (0 to 0.20) + MN	1440	341	147	593	1.26	7.1
**30**	BU	—	Beef	FI	TE (0 to 0.20)	19	65	551	636	1.31	—
**31**	BU	NE	Beef	GR/FI	TE (0 to 0.07) + MN	120	139	308	488	1.32	9.7
**32**	ST	—	Beef	FI	TE (0 to 0.09) + MN	443	120.1	222	359	1.21	8.4
**33**	BU	Mix	Beef	FI	TE (0 to 0.15) + MN	160	90	343	535	1.82	10.2
**34**	HE	AN	Beef	FI	TE (0 to 0.60)	110	91	185	273	1.34	9.1
**35**	Mix	BI	Beef	GR/FI	TE (0 to 0.20) + MN + VM	188	134	232	381	1.11	7.1
**36**	HE	HO	Dairy	GR	TE (0 to 0.30)	20	45	—	252	0.42	6.6
**37**	ST	Mix	Beef	GR/FI	TE (0 to 0.15) + MN	160	328	130	616	1.48	7.9
**38**	HE	HO	Beef	GR	TE (0 to 0.09) + MN	36	62	114	179	1.04	5
**39**	BU	Mix	Beef	GR/FI	TE (0 to 0.07) + Flavomycin	465	101	430	533	1.01	—
**40**	BU	NE	Beef	GR/FI	TE (0 to 0.07) + MN	1198	103	336	498	1.57	10.7
**41**	Mix	Mix	Beef	GR/FI	TE (0 to 0.08) + MN	173	100	245	359	1.16	7.3
**42**	BU	NE	Beef	FI	TE (0 to 0.08) + MN + VM	13967	121	387	606	1.81	10.1
**43**	BU	BR	Beef	GR/FI	TE (0 to 0.075) + MN + Vit E	294	104	353	487	1.3	9.8

1 References: 1 = Barajas et al. (2011b), 2 = Barajas et al. (2010), 3 = Barajas et al. (2011a), 4 = Mezzomo et al. (2011), 5 = Sartor Neto et al. (2011), 6 = Barajas et al. (2012b), 7 = Barajas et al. (2012a), 8 = Barajas et al. (2013), 9 = Cervantes et al. (2013), 10 = Montoya et al. (2013), 11 = Pasinato et al. (2012); internal report, 12 = Barajas et al. (2015), 13 = Mezzomo et al. (2016), 14 = Cabral et al. (2016), 15 = Rivera-Méndez et al. (2017), 16 = Ebert et al. (2017), 17 = Barrios (2017); thesis, 18 = Piñeiro-Vázquez et al. (2018), 19 = Tabke et al. (2017), 20 = Aboagye et al. (2018), 21 = Cunha et al. (2018); thesis, 22 = dos Anjos (2019); thesis, 23 = Martello et al. (2020), 24 = Norris et al. (2020), 25 = Demarco et al. (2021), 26 = Ferracini et al. (2024), 27 = Cidrini et al. (2022), 28 = Montano et al. (2022), 29 = Bowman et al. (2023), 30 = He et al. (2023), 31 = Magnani et al. (2023), 32 = Mango et al. (2024); internal report, 33 = Nascimento et al. (2024), 34 = Paz et al. (2007); internal report, 35 = Vidart et al. (2024); internal report, 36 = Schilling-Hazlett et al. (2023), 37 = Carvalho et al. (2024), 38 = Urrutia et al. (2023), 39 = Manella et al. (2024b), 40 = Manella et al. (2024a), 41 = Cabral et al. (2022); internal report, 42 = Manella and Desrues (2023); internal report, and 43 = Lacherre et al. (2024).

2 BU = bulls, HE = heifers, and ST = steers.

3 AN = Angus, BI = British breed, BR = Brahman, CO×BI = Continental × British crossbreds, HO = Holsteins, IN = Bos Indicus (not specified), Mix = multiple breeds, and NE = Nellore.

4 GR = growing and FI = finishing.

5 Cr = chromium, DFM = direct-fed microbials, MN =monensin, TE = tannin extract, TMP = TMP Protein Enhancer^®^ (Técnica Mineral Pecuaria, Mexico), TY = tylosin, VM = virginiamycin, YT = yeast, and ZIL = zilpaterol hydrochloride.

6 ADG = average daily gain, kg/d; DMI = dry matter intake, kg/d; DOF = days on feed (i.e., trial); fBW = final body weight, kg; iBW = initial body weight, kg; and n = sample size.

Table 1 summarizes additional treatments used in the studies, either in combination with TE or independently. These included feed additives such as urea, chromium, protein supplements, and direct-fed microbials; antibiotics such as monensin and virginiamycin; and growth-promoting implants such as zilpaterol hydrochloride. The dataset spanned multiple cattle breeds, including Nellore, Angus, Holsteins, and various crossbreds, across both growing and finishing phases. Animals were classified in the original publications as bulls (22 studies), steers (12 studies), heifers (6 studies), or mixed groups (3 studies). Most trials were conducted in feedlot settings; however, the dataset also included grazing studies and individual-pen trials. Outcomes included ADG (kg/d) and DMI (kg/d). G:F (g/kg) was calculated as G:F = (ADG × 1000)/DMI using recorded ADG and DMI values.

### Meta-analytic framework

***Meta-regression analysis.*** Meta-regression was conducted using the restricted maximum likelihood (REML) method. ADG (kg/d), DMI (kg/d), and G:F (g/kg) were modeled as outcomes against a tannin exposure moderator expressed as either TE (% of dietary DM) or TE intake (g/d). An unstructured variance-covariance matrix was assumed, and studies (PubID) were modeled as random effects. Where applicable, the number of animals per treatment group was used as a weight to account for sample size differences. Analyses were performed on the complete dataset and within subsets defined by TE dose range or the presence of additional feed additives and implants. The linear mixed-effects model was:


$$Y_{ij}\;=\;\beta_{0}+\;\beta_{1}X_{ij}+\;b_{0j}+\;b_{1j}X_{ij}+\;\varepsilon_{ij}$$

where, $Y_{ij}$ is the outcome variable (e.g., mean difference or effect size) for observation *i* in study *j*, $X_{ij}$ represents the moderator variable (e.g., treatment-related covariates), $\beta_{0}$ and $\beta_{1}$ are the fixed-effect coefficients (intercept and slope), $b_{0j}$ and $b_{1j}$ are the random effects for intercept and slope for study *j*, assumed to follow a bivariate normal distribution:


(b0jb1j) ∼ N((00), D)

where, $\varepsilon_{ij}\;∼\;N(0,\sigma^{2}{\omega_{ij}}^{-1})$ denotes the residual error term, where $\omega_{ij}$ is the weight for each observation, defined as the sample size (e.g., number of animals per treatment group). This structure allowed both intercepts and slopes to vary by study, thereby accounting for between-study heterogeneity, while weighting addressed heteroscedasticity.

To evaluate potential curvature, a quadratic extension was considered after mean-centering the moderator for numerical stability, ${\tilde{X}}_{ij}=X_{ij}-\tilde{X}$.


$$Y_{ij}\;=\;\beta_{0}+\;\beta_{1}{\tilde{X}}_{ij}+\;{\beta_{1}{{\tilde{X}}_{ij}}^{2}+b}_{0j}+\;b_{1j}{\tilde{X}}_{ij}+\;\varepsilon_{ij}$$

Random effects were specified for the intercept and linear slope; random quadratic terms were not included. Selection between the linear and quadratic fixed-effect structures was carried out by first fitting both models under maximum likelihood and comparing Akaike information criterion (AIC) values; the model with the lower AIC was preferred.

For ADG, the TE dose–response relationship was also evaluated with or without monensin supplementation. The presence or absence of monensin supplementation was included as a fixed-effect factor, and its interactions with the linear and quadratic TE terms were assessed in mixed-effects models. Linear and quadratic models with and without these interaction terms were fitted under maximum likelihood, using the same random-effects and weighting structure described above, and compared by likelihood ratio tests. Because these tests did not provide strong evidence that either the slope or curvature of the ADG response differed according to monensin supplementation status (*P *= 0.13, data not shown), subsequent ADG-based dose–response procedures that depended on the meta-regression (e.g., broken-line analysis) were conducted on the pooled dataset without stratification by monensin inclusion.

Predicted values from the meta-regression were subsequently used for additional statistical testing. Specifically, one-sample *t*-tests were applied to evaluate whether the predicted performance metrics (e.g., ADG, DMI, G:F) at given TE inclusion levels differed significantly from baseline estimates derived from the model. This allowed for formal significance testing of model-derived predictions while accounting for between-study variability. Equivalent procedures were implemented for all primary performance outcomes.

***Meta-analyses.*** Random-effects meta-analyses were conducted to estimate the overall effect size across studies. The model was:


$$Y_{i}=\;\theta +\;u_{i}+\;\varepsilon_{i}$$

where, $Y_{i}$ denotes the effect size estimate from the i-th study, θ is the overall average effect (fixed effect), $u_{i}\;∼\;N(0,\;\tau^{2})$is the study-specific random effect capturing between-study heterogeneity and $\varepsilon_{i}\;∼\;N(0,\;{\sigma^{2}}_{i})$ is the within-study sampling error. The study-specific random effects were assumed to follow a normal distribution with a mean of zero and variance $\tau^{2}$, while the residual errors were modeled with variances corresponding to the study-level sampling error.

Heterogeneity among studies was assessed using several standard statistics. Cochran’s Q statistics was calculated to test for the presence of heterogeneity, defined as:


$$Q=\sum_{i=1}^{k}w_{i}{\left(y_{i}-{\bar{y}}_{w}\right)}^{2}$$

where, k is number of studies, $w_{i}$ are the inverse-variance weights, $y_{i}$ are the observed effect sizes, and ${\bar{y}}_{w}$ is the weighted mean effect size. Based on Q, standard heterogeneity statistics, including $\tau^{2}$ (between-study variance), $I^{2}$ (proportion of total variability attributable to heterogeneity), $H^{2}$ (relative impact of heterogeneity), and $R^{2}$ (proportion of explained heterogeneity), were used to evaluate model performance.

The standardized mean difference (SMD) was calculated as $(m_{1i}-m_{2i})/{sp}_{i}$, where ${sp}_{i}$ is the pooled standard deviation of the two groups. The SMD metric was used to synthesize studies reporting outcomes on different scales. Additional covariates could be added to the model; in our case, PubID was treated as a random effect to account for clustering. Confidence and prediction intervals were also generated from the forest plot (Cairns et al. 2022).

### Post-meta-analyses evaluation

***Broken-line analysis.*** A broken-line analysis was used to identify potential breakpoints in the relationship between TE dosage and ADG. The analysis was performed on ADG values adjusted for study-level effects using the meta-regression model described above. After estimating the breakpoint, the analysis focused on the ascending (pre-breakpoint) region by retaining the observations at or below the breakpoint and fitting linear and quadratic models to that segment; model preference was determined by comparing AIC values.

***Response surface regression.*** A response surface regression was conducted to explore the relationship among crude protein intake (CPI, kg/day), TE supplementation (g/day), and ADG (kg/day). Study-level variability was modeled by including random effects for both intercept and slope across studies (PubID), assuming an unstructured variance-covariance matrix. The model was initially fitted using the REML method. When convergence failed, maximum likelihood estimation was used instead. The number of animals per treatment group was applied as an inverse-variance weight to account for heteroscedasticity. This approach enabled a more robust analysis of variance components across heterogeneous data. Extended iteration controls were used to monitor model convergence and performance.

### Nonparametric analysis

Nonparametric analyses were conducted specifically on the ADG variable to evaluate the categorical association between TE supplementation and growth response. Contingency tables (Horton and Kleinman 2010) and χ^2^ tests (Agresti 2018) were utilized to explore the independence and interaction of variables. In particular, we examined whether the observed ADG differences between treatment and control groups were influenced by the level of TE supplementation and the presence of monensin, a commonly used ionophore. The analyses were stratified in two key ways. First, treatment groups were separated based on the presence or absence of monensin to assess potential interactive effects between TE and monensin. For this comparison, other feed additives and implants were not included as additional factor in this analysis to keep the 2 × 2 design interpretable; therefore, this analysis evaluates TE–monensin association only and does not rule out confounding by co-additives present in the underlying studies. Second, the effects of TE were further examined across different inclusion levels, using TE thresholds at 0.08%, 0.15%, and 0.30% of dietary DM. These TE inclusion thresholds were chosen to represent low, moderate, and high exposure levels and to reflect the commonly reported TE inclusion ranges in the literature and in our dataset (0.08, 0.15, and 0.30% of dietary DM), which showed clear clustering around these values. For each threshold, 2 × 2 contingency tables were constructed in which the first dimension reflected whether the treatment effect was statistically distinguishable from the control (*P *< 0.10) or not (*P *≥ 0.10), and the second was the TE dose category (below vs. above the threshold). These tables allowed evaluation of whether higher TE inclusion was more likely to result in a significant ADG response. To test for independence within these categorical data, Fisher’s χ^2^ test and McNemar’s χ^2^ test were applied. Conditional independence testing with 5,000 permutation distributions was used to assess additional relationships between factors (Zeileis et al. 2007). Odds ratios were calculated to quantify the relative likelihood of observing an ADG effect in relation to TE inclusion level. Mosaic plots were used to visualize the contingency table data (Hartigan and Kleiner 1984; Friendly 1994; Emerson 1998; Meyer et al. 2006; Friendly and Meyer 2016). In these plots, cell size corresponded to frequency counts, with width and height reflecting marginal row and column probabilities. The color and outline of each cell represented standardized Pearson residuals: blue with solid outlines for positive deviations, and red with dashed outlines for negative deviations. Color intensity indicated the level of statistical significance, with darker colors representing *P *< 0.001 and lighter colors denoting *P *< 0.01.

### Software and visualization

All analyses were conducted in R software (version 4.5.0; R Core Team 2025). Meta-analyses and forest plots were generated using the *metafor* package (Viechtbauer 2010), and meta-regression models were fitted using the *nlme* package (Pinheiro et al. 2017). Additional analyses used the *segmented* package for broken-line models (Muggeo 2003; Muggeo 2008). Figures were produced using base R graphics (plot function) and the *ggplot2* package (Wickham 2016; Wickham and Grolemund 2017), and three-dimensional graphics were generated in Mathematica (version 14; Wolfram 2003).

## Results

The dataset for this meta-analysis comprised 70 records from bulls, encompassing 17,734 animals across 23 studies, and 61 records from steers, encompassing 3,775 animals across 13 studies (Table 2 and Figures S1 to S4). In total, the database included 59 control groups and 98 treatment groups. The dietary inclusion levels of TE varied considerably, ranging from 0.0% to 4.5% of dietary DM.

**Table 2:** Descriptive statistics of tannin extract dose and performance outcomes by treatment and gender class.

Items^1^	Treatment	Gender	No. of studies	No. of animals	Mean	S.D.	Min	Max
**TE dose**	Control	Bull	23	14,070	—	—	—	—
		Steer	13	1,642	—	—	—	—
		Heifer	7	265	—	—	—	—
		Mix	2	237	—	—	—	—
	TE	Bull	23	3,664	0.198	0.1270	0.063	0.400
		Steer	7	2,133	0.643	0.8480	0.075	4.500
		Heifer	2	427	0.884	1.2400	0.075	4.000
		Mix	13	239	0.215	0.0218	0.200	0.231
**ADG**	Control	Bull	21	14,054	1.31	0.416	0.28	1.97
		Steer	11	1,546	1.32	0.335	0.72	2.08
		Heifer	6	260	1.02	0.327	0.37	1.32
		Mix	2	237	1.15	0.059	1.11	1.19
	TE	Bull	21	3,648	1.43	0.378	0.30	2.15
		Steer	11	1,949	1.39	0.374	0.67	2.14
		Heifer	6	407	1.03	0.437	0.39	1.45
		Mix	2	239	1.18	0.106	1.11	1.26
**DMI**	Control	Bull	20	13,796	9.1	1.40	6.9	12.0
		Steer	13	1,624	7.6	2.29	4.0	11.3
		Heifer	7	265	7.0	1.96	4.6	10.2
		Mix	2	237	6.8	0.40	6.5	7.0
	TE	Bull	20	3,390	9.1	1.57	6.3	12.1
		Steer	13	2,097	8.1	2.49	4.0	11.8
		Heifer	7	427	7.3	1.50	5.3	10.3
		Mix	2	239	6.7	0.57	6.3	7.1
**G:F**	Control	Bull	9	12,827	149	26.2	100	181
		Steer	7	340	164	47.7	92	256
		Heifer	4	165	164	45.6	100	208
		Mix	0	—	—	—	—	—
	TE	Bull	9	1,926	153	27.9	90	182
		Steer	7	588	155	48.2	92	257
		Heifer	4	279	137	48.3	80	208
		Mix	0	—	—	—	—	—

1 TE, tannin extract, % of dietary DM; ADG, average daily gain, kg/d; DMI, dry matter intake, kg/d; G:F, gain:feed, g/kg.

2 S.D., standard deviation; Min, minimum; Max, maximum.

**Figure 1:**
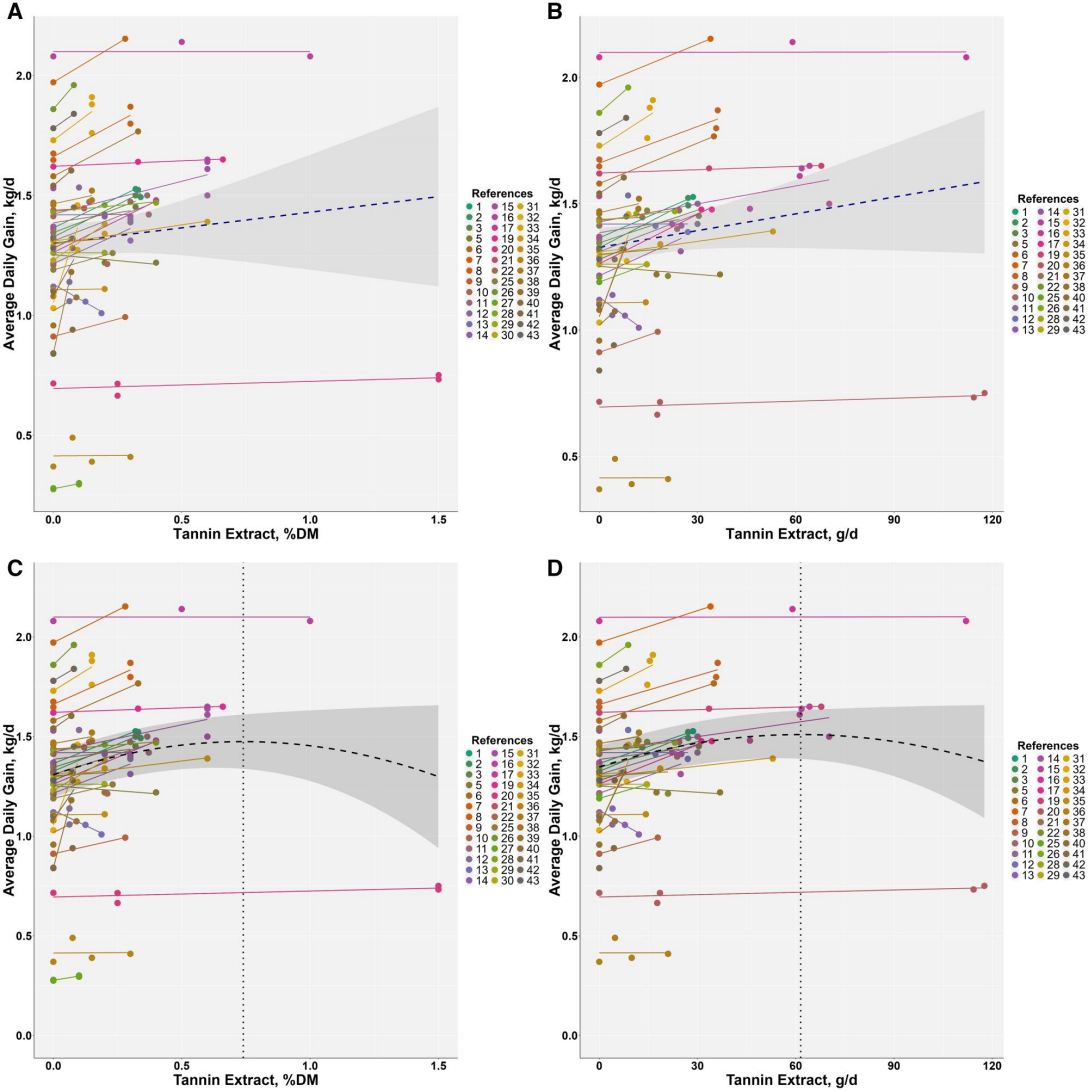
Meta-regression for average daily gain (ADG, kg/d) versus tannin extract (% of dietary DM) and tannin extract intake (g/d). The relationship is shown using both linear (A and B) and quadratic (C and D) models. The following equations represent the relationships after adjusting for the random effects of studies: (A) ADG = 1.317 ± 0.0051 + 0.250 ± 0.0411 × TE (% of dietary DM) (r^2^ = 0.23, *P *< 0.001, AIC = 0.26); (B) ADG = 1.3511 ± 0.00447 +0.0034 ± 0.00040 × TE (g/d) (r^2^ = 0.38, *P *< 0.001, AIC = −27.49); (C) ADG = 1.308 ± 0.0051 + 0.450 ± 0.0649 × TE—0.304 ± 0.0789 × TE^2^ (% of dietary DM) (r^2^ = 0.31, *P *< 0.001, AIC = −4.67, vertex: 0.740, 1.475); (D) ADG = 1.347 ± 4.688 × 10^-^³ + (5.292 × 10^-^³ ± 7.365 × 10^−4^) × TE − (4.302 × 10^−5^ ± 1.086 × 10^−5^) × TE^2^ (g/d) (r^2^ = 0.37, *P *< 0.001, AIC: −31.41, vertex: 61.5, 1.510).

***Average daily gain***. A total of 124 treatment records from 40 studies (22,340 animals) were used to analyze ADG (Table 2 and Figure S2). ADG values ranged from 0.275 to 2.15 kg/d, with most data from bulls. Meta-analysis indicated that TE accounted for 9.97% of the total variance in ADG and that residual heterogeneity was significant (*P *< 0.001) (Figure S5). Study-adjusted meta-regressions are summarized in Figure 1. When TE was expressed as a percentage of dietary DM, ADG increased by approximately 0.25 kg/d per 1 percentage unit increase in TE inclusion (r^2^ = 0.23, *P *< 0.001) (Figure 1A). When TE was expressed as intake in grams per day, ADG increased by approximately 0.0034 kg/d per gram (r^2^ = 0.38, *P *< 0.001) (Figure 1B). Quadratic models had vertices at 0.74% of dietary DM with ADG approximately 1.474 kg/d (r^2^ = 0.31, *P *< 0.001; AIC = −4.67 relative to the linear model) (Figure 1C) and at 61.5 g/day with ADG approximately 1.51 kg/day (r^2^ = 0.37, *P *< 0.001; AIC = −31.41 relative to the linear model) (Figure 1D). Overall, AIC favored the quadratic forms over the linear alternatives, although the advantage was modest.

**Figure 2:**
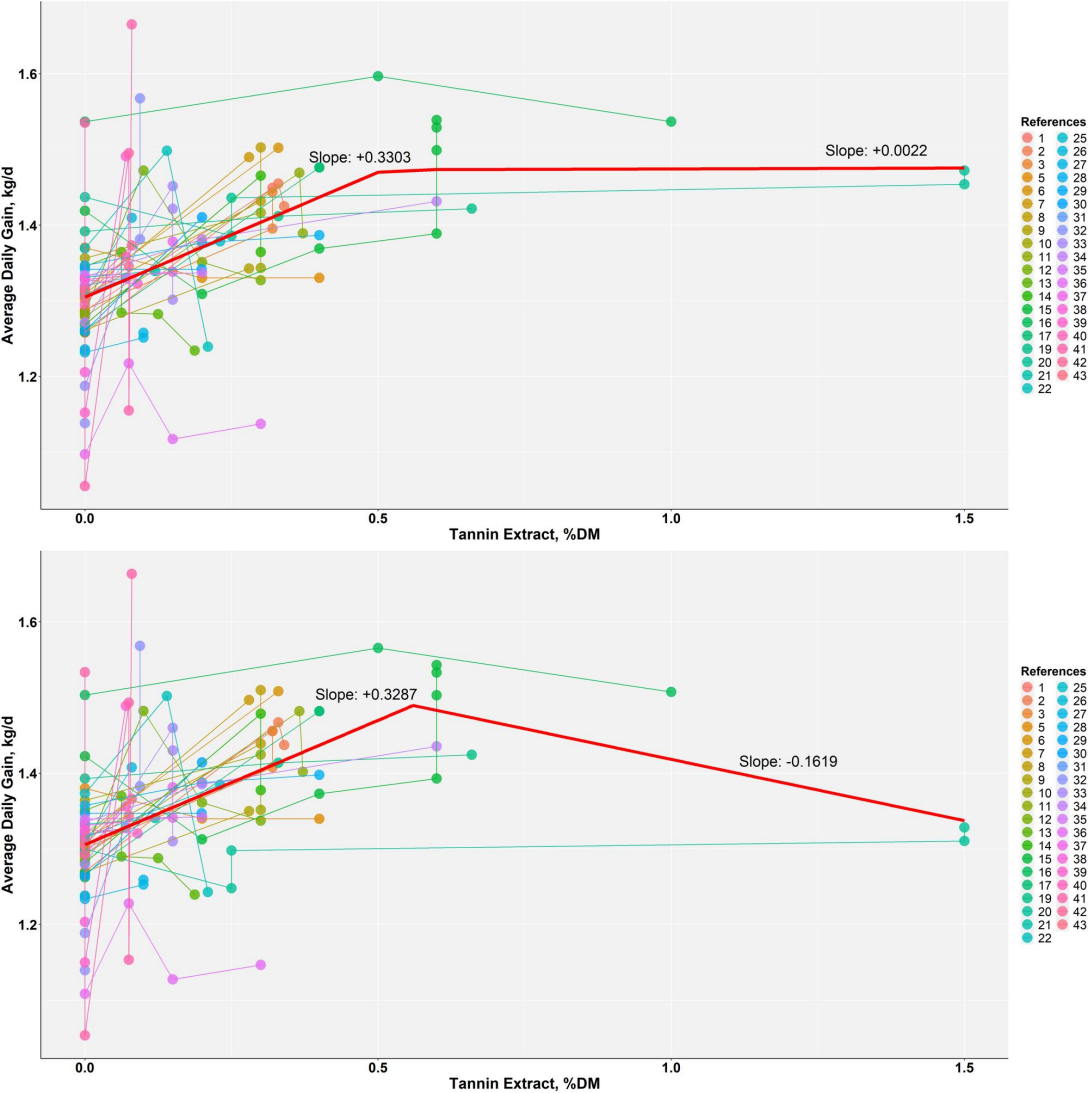
Broken-line analysis of the average daily gain (ADG, kg/d) on tannin extract (% of dietary DM), not weighted (upper panel) and weighted (study-adjusted, bottom panel) by sample size (number of animals per treatment). The breakpoint was 0.51% of dietary DM in the upper panel and 0.56% of dietary DM in the bottom panel.

**Figure 3:**
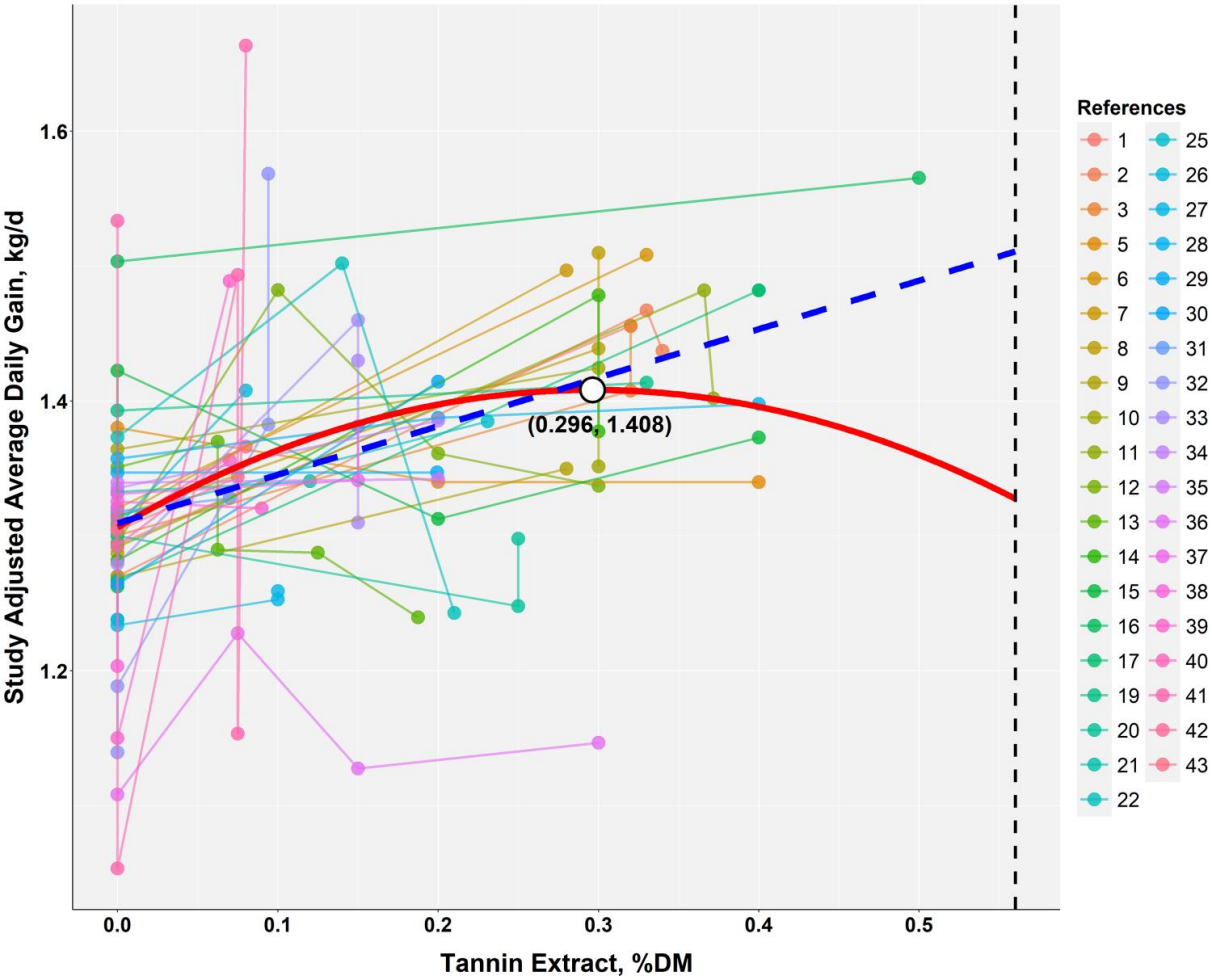
Comparison of linear and quadratic fits for study-adjusted average daily gain (ADG, kg/d) on tannin extract using left-of-breakpoint data (≤ 0.56% of dietary DM, n = 115). The linear equation was ADG = 1.309 ± 0.0053 + 0.360 ± 0.0556 × TE (% of dietary DM) (r^2^ = 0.27, *P *< 0.001, AIC = −161.4). The quadratic equation was ADG = 1.306 ± 0.0054 + 0.687 ± 0.1658 × TE − 1.160 ± 0.5542 × TE^2^ (% of dietary DM) (r^2^ = 0.30, *P *< 0.001, AIC = −163.8). This curve attains a maximum at TE = 0.296% of dietary DM with ADG = 1.408 kg/d.

A broken-line analysis for TE as a percentage of dietary DM identified a breakpoint at 0.51% dietary DM in the unweighted model and at 0.56% of dietary DM in the model weighted by animals per treatment (Figure 2). Using the weighted breakpoint 0.56% of dietary DM and fitting only the left-of-breakpoint data ≤ 0.56% of dietary DM, n = 115, the study-adjusted linear model estimated an increase of approximately 0.36 kg/d per 1 percentage unit increase in TE inclusion (r^2^ = 0.27, *P *< 0.001) (Figure 3). A quadratic fit on the same subset had a slightly lower AIC (AIC −163.8 vs. −161.4; ΔAIC = 2.4) and reached a local maximum at 0.296% of dietary DM with ADG approximately 1.408 kg/d (r^2^ = 0.30, *P *< 0.001) (Figure 3). Within this lower TE range, the estimated increments in ADG decreased near the breakpoint, and the fitted curve approached a maximum (Figure 3).

**Figure 4:**
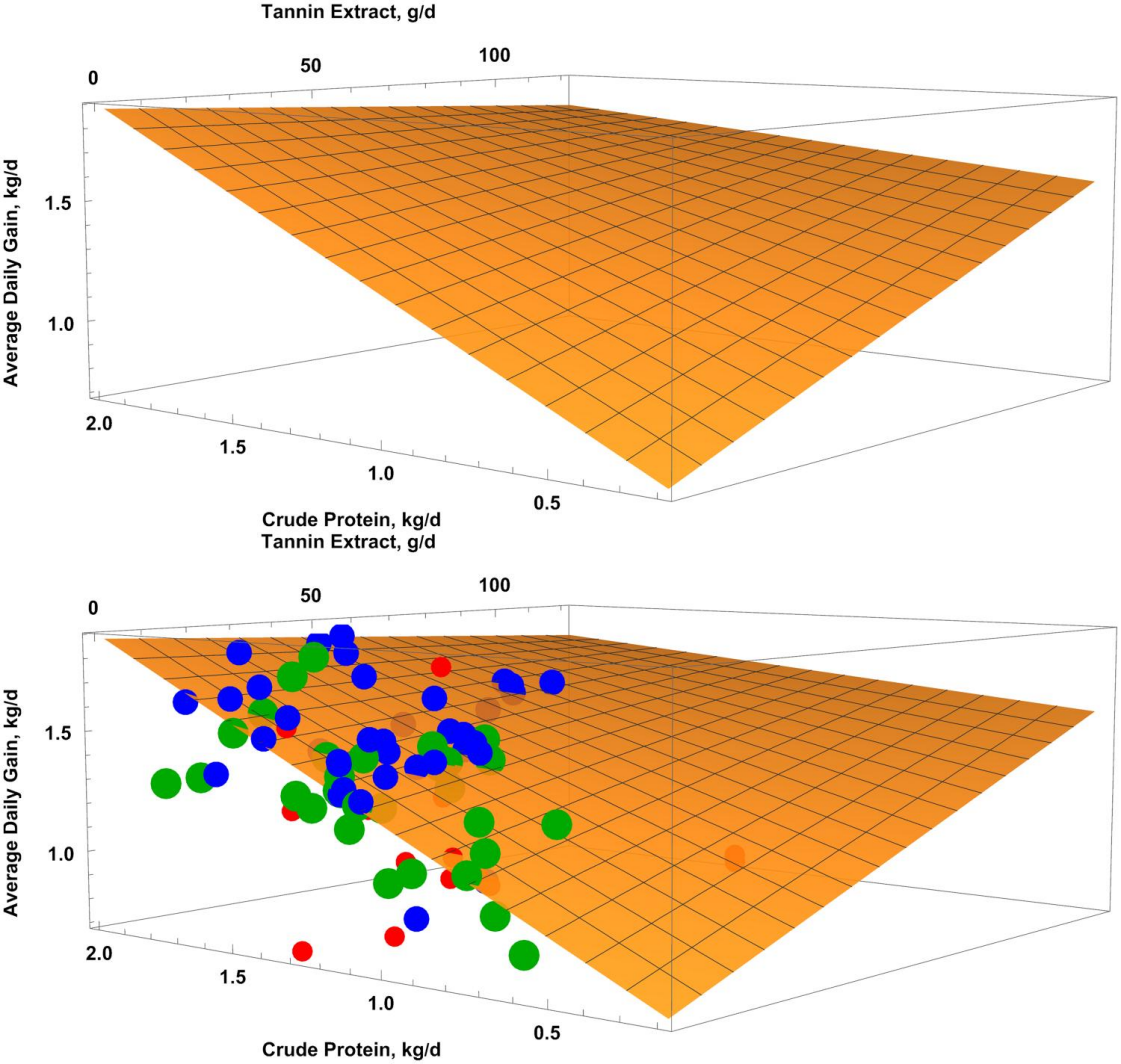
Response surface plot of crude protein intake and tannin extract intake on average daily gain (without and with the points) and not weighted by sample size. The equation was ADG = 0.5685397 + 0.0079425 × TEI + 0.6566805 × CPI—0.0044771 × TEI × CPI (*P*-value = 0.0525, AIC = –68). Red dots mean studies with fewer than 20 animals per treatment, blue dots mean studies with more than 20 but fewer than 50 animals per treatment, and green dots mean studies with more than 50 animals per treatment. The 20 and 50 represent the 33% and 66% quartiles of the data.

**Figure 5:**
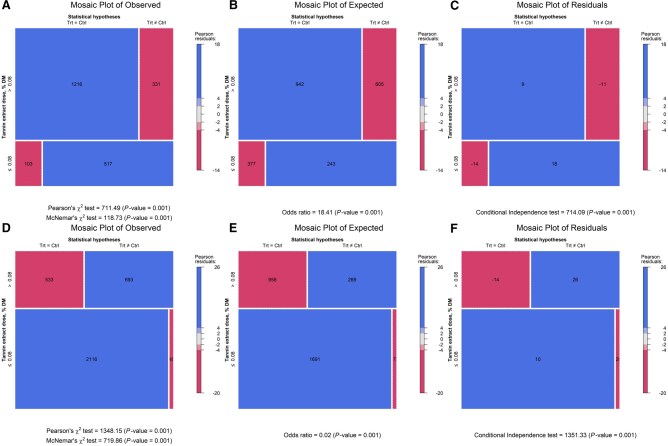
Mosaic plots of contingency tables illustrating the effects of tannin extract (TE) with (panels A, B, and C) and without (panels D, E, and F) monensin supplementation on average daily gain (ADG) at a TE inclusion level of 0.08% of dietary DM.

**Figure 6:**
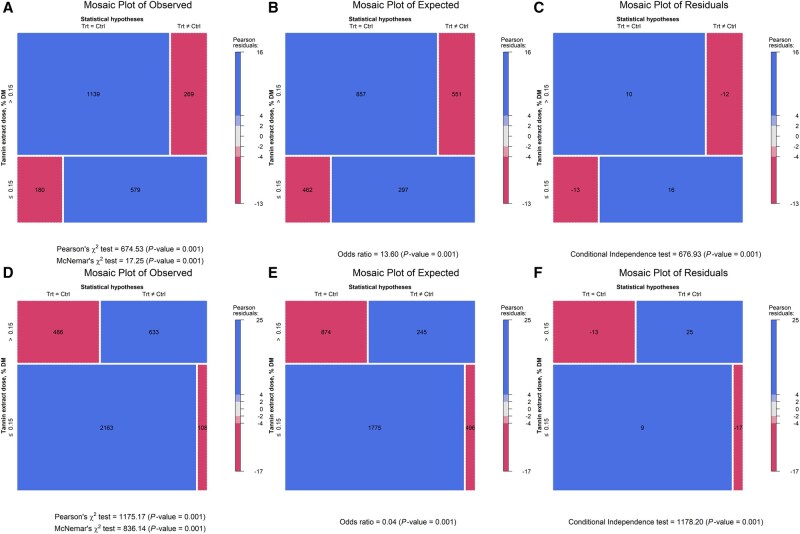
Mosaic plots of contingency tables illustrating the effects of tannin extract (TE) with (panels A, B, and C) and without (panels D, E, and F) monensin supplementation on average daily gain (ADG) at a TE inclusion level of 0.15% of dietary DM.

**Figure 7:**
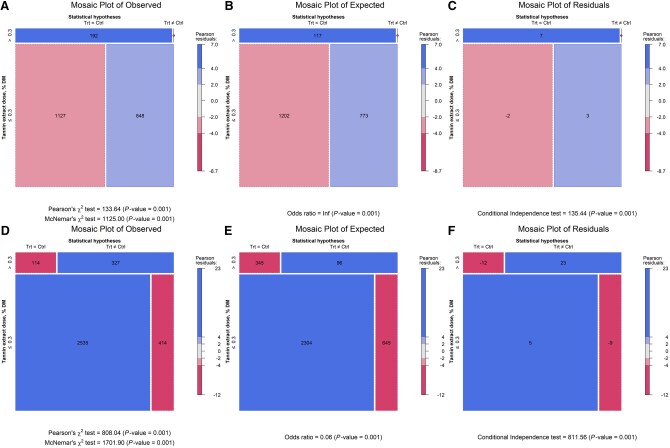
Mosaic plots of contingency tables illustrating the effects of tannin extract (TE) with (panels A, B, and C) and without (panels D, E, and F) monensin supplementation on average daily gain (ADG) at a TE inclusion level of 0.30% of dietary DM.

The response surface regression showed similar patterns in both the weighted and unweighted models. The regression for the unweighed model was: ADG = 0.5685397 + 0.0079425×TEI + 0.6566805×CPI—0.0044771×TEI×CPI (*P*-value = 0.0525, AIC = −68), and the weighed model was ADG = 0.3798324 + 0.0074662×TEI + 0.8100061×CPI—0.0042303×TEI×CPI (*P*-value = 0.1272, AIC = −28.4). The unweighted model had the lower AIC (−68 vs. −28.4) and is shown in Figure 4. The response surface plot revealed that when CPI was ∼0.5 kg/d, ADG approached zero without TE. In contrast, higher TE levels supported ADG above 1.0 kg/d. Contingency analyses further examined interactions between TE and monensin at TE thresholds of 0.08%, 0.15%, and 0.30% of dietary DM (Figures 5–7). At all thresholds, the proportion of animals with a significant ADG response was higher when TE was combined with monensin. For example, at the 0.08% of dietary DM threshold, 21% of animals receiving TE alone showed a significant response, consistent with a stronger TE-associated ADG response (56.5%) in monensin-containing studies, suggesting a potential synergistic effect though some studies had additional feed additives.

**Figure 8:**
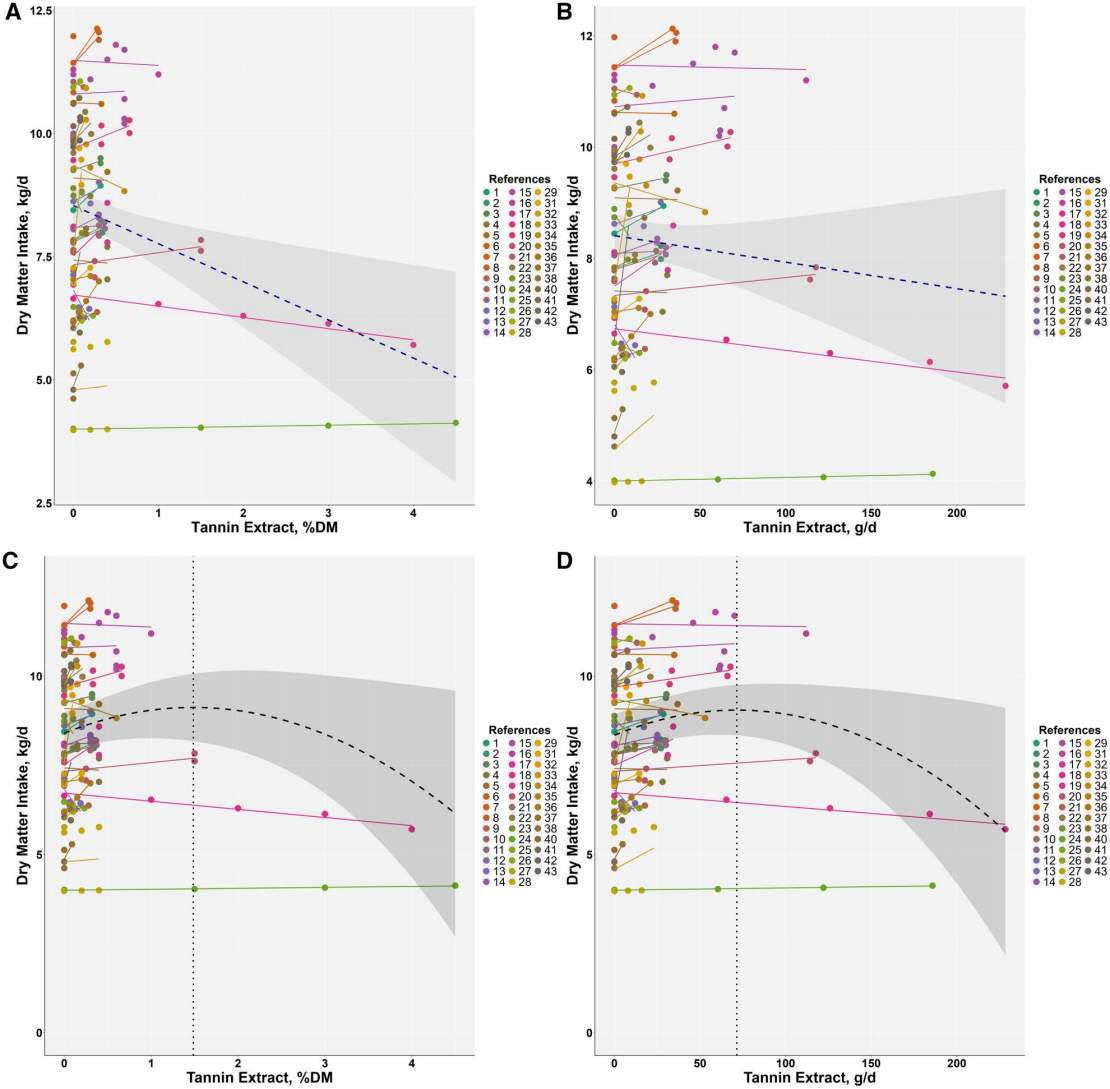
Meta-regression for dry matter intake (DMI, kg/d) versus tannin extract (% of dietary DM) and tannin extract intake (g/d). The relationship is shown using both linear (A and B) and quadratic (C and D) models. The following equations represent the relationships after adjusting for the random effects of studies: (A) DMI = 8.4 ± 0.0311 + 0.688 ± 0.1662 × TE (% of dietary DM) (r^2^ = 0.11, *P *< 0.001, AIC = 528.9); (B) DMI = 8.389 ± 0.0319 + 0.007 ± 0.0024 × TE (g/d) (r^2^ = 0.06, *P *= 0.004, AIC = 529.2). (C) DMI = 8.405 ± 0.0328 + 0.973 ± 0.2846 × TE—0.327 ± 0.0982 × TE^2^ (% of dietary DM) (r^2^ = 0.08, *P *= 0.002, AIC = 528.5, vertex: 1.485, 9.127); (D) DMI = 8.347 ± 0.0325 + 0.020 ± 0.0039 × TE—0.0001 ± 0.00004 × TE^2^ (g/d) (r^2^ = 0.15, *P *< 0.001, AIC = 523.4, vertex: 71.5, 9.053).

***Dry matter intake***. The DMI was assessed using 143 treatment records from 42 studies (Table 2 and Figure S4), with values ranging from 4.62 to 12.1 kg/d. The meta-analysis indicated that TE supplementation accounted for 15.11% of the total variance in DMI (Figure S6). In the study-adjusted linear models, DMI increased by approximately 0.69 kg/d per 1 percentage unit increase in TE inclusion (r^2^ = 0.11, *P *< 0.001) and by approximately 0.007 kg/d per gram of TE intake (r^2^ = 0.06, *P *= 0.004) (Figure 8A and B). Quadratic models had vertices at 1.485% of dietary DM with DMI approximately 9.13 kg/d (r^2^ = 0.08, *P *= 0.002) (Figure 8C) and at 71.5 g/d with DMI approximately 9.05 kg/d (r^2^ = 0.15, *P *< 0.001) (Figure 8D). For both TE and TE intake, the quadratic models had lower AIC than the linear models (ΔAIC = 0.4 for percentage of DM; ΔAIC = 5.8 for TE intake). However, the explained variance was low, as indicated by the r^2^ values across models.

**Figure 9:**
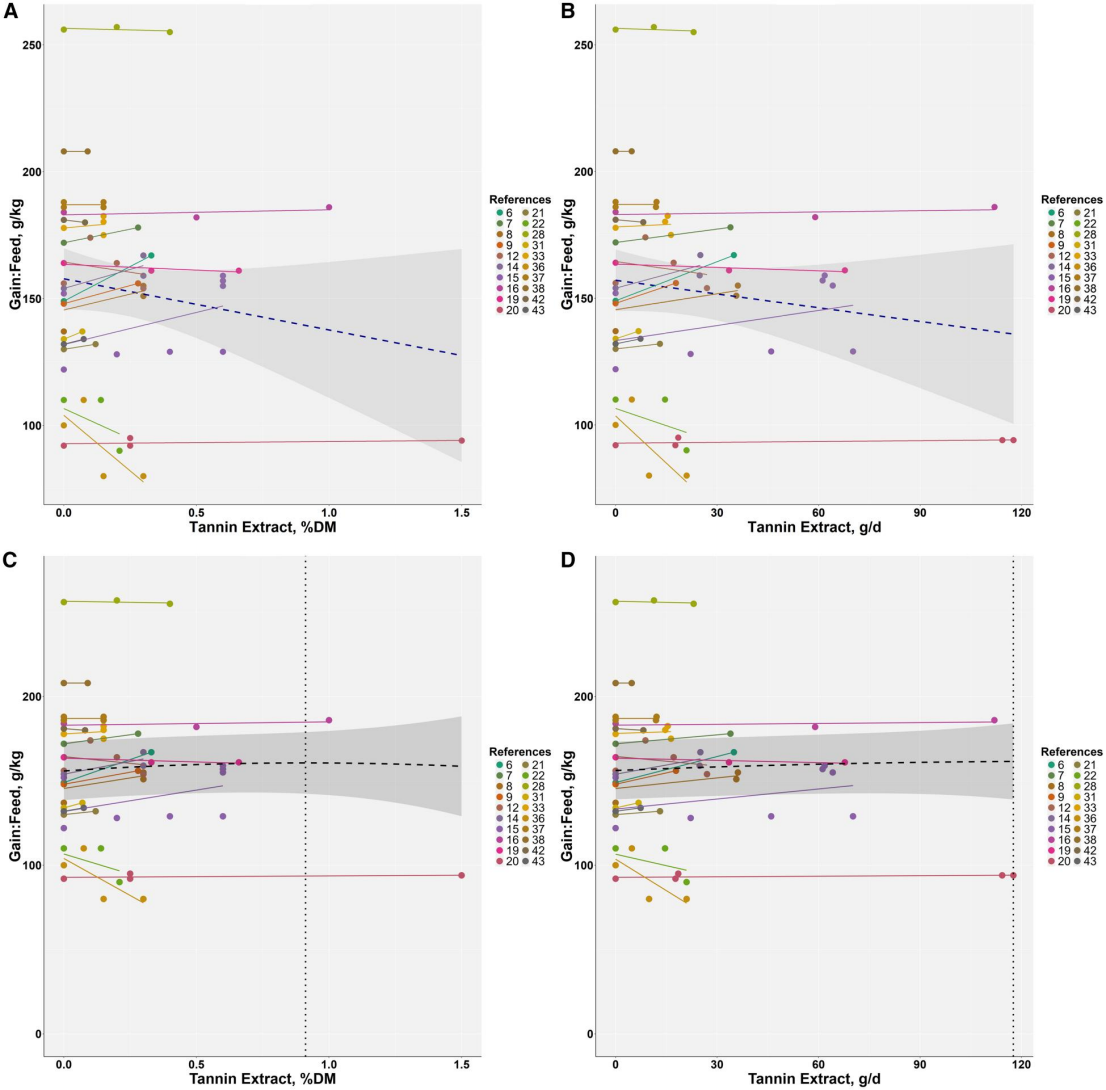
Meta-regression for gain:feed (G:F, g/kg) versus tannin extract (% of dietary DM) and tannin extract intake (g/d). The relationship is shown using both linear (A and B) and quadratic (C and D) models. The following equations represent the relationships after adjusting for the random effects of studies: (A) G:F = 156.230 ± 0.2740 + 5.576 ± 2.4190 × TE (% of dietary DM) (r^2^ = 0.08, *P *= 0.024, AIC = 555.1); (B) G:F = 156.2367 ± 0.27482 +0.0593 ± 0.02611 × TE (g/d) (r^2^ = 0.07, *P *= 0.027, AIC = 555.1). (C) G:F = 155.86 ± 0.2657 + 10.52 ± 4.243 × TE—5.77 ± 4.298 × TE^2^ (% of dietary DM) (r^2^ = 0.11, *P *= 0.023, AIC = 556.7, vertex: 0.912, 160.651); (D) G:F = 156.2 ± 0.2846 + 0.079 ± 0.0551 × TE—2.867 × 10^−4^ ± 7.099 × 10^−4^ × TE^2^ (g/d) (r^2^ = 0.08, *P *= 0.077, AIC = 557.1, vertex: 138.485, 161.665).

***Gain:Feed***. The G:F was analyzed using 66 treatments from 20 studies in which both ADG and DMI were reported simultaneously (Table 2 and Figure S5). G:F values ranged from 80 to 257 g/kg. Meta-analysis (Figure S7) indicated that the model did not explain any variance in G:F (r^2^ = 0.00), and substantial heterogeneity was observed. In the study-adjusted linear fits, G:F increased by approximately 5.6 g/kg per 1 percentage unit increase in TE inclusion (r^2^ = 0.08, *P *= 0.024) and by approximately 0.059 g/kg per gram of TE intake (r^2^ = 0.11, *P *= 0.023) (Figure 9A and B). Quadratic fits had vertices at 0.912% of dietary DM with G:F approximately 160.7 g/kg (r^2^ = 0.08, *P *= 0.077) and at 138.5 g/d with G:F approximately 161.7 g/kg (r^2^ = 0.08, *P *= 0.027) (Figure 9C and D). Model selection criteria indicated only small differences relative to the linear models (ΔAIC ≤ 2); however, the explained variance was low, as indicated by the r^2^ values.

## Discussion

Understanding how TE influences ruminant growth performance is important for exploring strategies to improve nutrient utilization and optimize production outcomes in livestock systems. Although many studies have evaluated the effects of CT and HT tannins on growth performance, the findings have been inconsistent. These inconsistencies are primarily due to variations in tannin sources, concentrations, animal species, dietary composition, and experimental conditions (Patra and Saxena 2011). Therefore, this study aimed to overcome these limitations by developing a comprehensive database and conducting a meta-analysis to evaluate the overall effects of TE, both when used independently and when applied in combination with other feed additives, on growth performance in growing and finishing cattle.

### Physiological mechanisms and overall performance

After adjusting for variance due to study effects, the meta-regression showed that TE supplementation was statistically associated with improvements in ADG, DMI, and FE. This suggests that TE has a consistent, though context-sensitive, positive effect on growth performance. This indicates that the performance gain is more likely due to improved nutrient utilization efficiency rather than increased feed intake. This interpretation is supported by the fact that the proportional gains in ADG were more pronounced than those in DMI. Tannins influence several ruminal processes that may underlie the observed improvements in growth performance. Notably, tannin supplementation has been shown to suppress protozoal populations, thereby mitigating predation pressure on fibrolytic bacteria and disrupting interspecies hydrogen transfer pathways that facilitate methanogenesis (Patra and Saxena 2009; Díaz Carrasco et al. 2017). In this context, the resulting reduction in enteric methane emissions can be interpreted as a mechanism of energy conservation, increasing the proportion of dietary energy retained by the host. Furthermore, tannins form complexes with dietary proteins, thereby limiting their degradation in the rumen and increasing the outflow of ruminal undegradable protein to the small intestine (Mezzomo et al. 2015; Loregian et al. 2023). This shift is likely to enhance post-ruminal amino acid absorption and nitrogen utilization, particularly when rumen-degradable protein supply is not limiting. In addition, tannin-induced alterations in microbial composition may promote fermentation profiles that favor propionate production and reduce ammonia accumulation (Min et al. 2003; Mueller‐Harvey 2006). These physiological shifts suggest that the gain in ADG is driven by improved metabolic efficiency rather than increased DMI.

### Non-linear dose-response relationships

A key finding of this study is that the relationship between TE supplementation and growth performance is nonlinear. Both broken-line and quadratic regression models provided a better fit to the ADG data than a simple linear model, indicating a dose-dependent response. The broken-line analysis identified a breakpoint at approximately 0.56% of dietary DM. To more precisely define the optimal point within this responsive range, a quadratic model was fitted to the data below this breakpoint. This refined analysis identified the vertex (i.e., maximum point) at 0.296% of dietary DM, corresponding to the predicted maximum ADG before the onset of diminishing returns. This provides a more precise target for optimization, indicating that while benefits accrue up to 0.56% of dietary DM, the physiological peak effect is likely achieved at a considerably lower inclusion level of around 0.30% of dietary DM. At moderate levels, TE may exert favorable impacts on nutrient metabolism and protein utilization, whereas excessive inclusion can suppress fermentation or reduce palatability, thereby diminishing intake and performance (Patra and Saxena 2011; Yanza et al. 2021). Notably, several studies within our dataset also showed neutral or negative responses at the lowest and highest tested doses, underscoring the need for dose precision.

However, it is important to distinguish between the modeled physiological optimum and the effective dose in production settings. Several recent in vivo trials have reported measurable improvements at ≤0.10–0.15% of dietary DM, and in some cases at approximately 0.08% of dietary DM, particularly in high-concentrate finishing diets and when co-fed with monensin (Ferracini et al. 2024; Manella et al. 2024a; Nascimento et al. 2024). Mechanistically, such low-dose responses are plausible if tannins primarily enhance nitrogen utilization and subtly shift fermentation toward greater propionate production without eliciting the anti-nutritional effects observed at higher inclusion levels. More recently, an in vitro study using a mixed CT and HT extracts also supported the efficacy of low doses by reporting that methane production was minimized at a TE inclusion rate of 0.18% of dietary DM, suggesting the greatest potential for methane mitigation at this dose (Adams et al. 2025). In our dataset, contingency analyses at the ≤0.08% of dietary DM stratum showed a markedly higher frequency of a positive ADG response when tannins were co-fed with monensin, with 517 of 620 animals (83.4%), than without monensin, with 48 of 2,164 animals (2.2%).

### Context-dependent efficiency and interactions

Despite these dose-response relationships, the explanatory power of TE supplementation across studies remained limited, with TE accounting for approximately 10% of the total variance in ADG. Most of the residual variability is likely attributable to between-study heterogeneity, including differences in basal diet composition, TE source, and interacting management factors. These findings align with previous reviews highlighting that the response to tannin supplementation is influenced mainly by the chemical structure of tannins, their concentration, the composition of the basal diet, delivery method, and management practices (Patra and Saxena 2011; Orzuna-Orzuna et al. 2021). These findings are not unique to beef cattle. Similar inconsistencies have been reported in dairy cows (Herremans et al. 2020) and small ruminants (Al Rharad et al. 2025), where tannin supplementation affected nitrogen metabolism, feed utilization, and productivity in highly variable ways, depending on factors such as tannin characteristics and dietary context.

Our analysis revealed that TE efficacy is modulated by specific dietary interactions. A response surface regression indicated that the TE-associated ADG response tended to be larger at crude protein intake. This interaction suggests that TE may enhance nitrogen utilization efficiency by reducing ruminal protein degradation and increasing the supply of bypass protein available for post-ruminal absorption (Tedeschi and Fox 2020). While this interpretation is physiologically plausible, it should be noted that few studies have systematically examined the interaction between dietary protein concentration and the effects of tannin supplementation under controlled conditions. In most existing studies (e.g., Mezzomo et al. 2015; Ebert et al. 2017; Tabke et al. 2017; Norris et al. 2020; Montano et al. 2022; Carvalho et al. 2024), tannin levels were varied while protein intake remained constant, making it difficult to isolate the influence of protein availability on tannin efficacy. Nevertheless, under conditions of limited protein supply, TE’s capacity to preserve dietary nitrogen appears to confer a compensatory advantage that supports continued growth.

Furthermore, the presence of ionophores appears to influence the effective dosing range of TE. Nonparametric contingency analysis indicated that the likelihood of a positive response in ADG was significantly greater when TE was combined with monensin, particularly when TE inclusion exceeded 0.15% of dietary DM. This outcome may reflect a complementary mode of action: monensin improves energy metabolism by modulating ruminal fermentation and increasing propionate production (Van Maanen et al. 1978; Armentano and Young 1983), while TE enhances nitrogen utilization by reducing ruminal protein degradation and ammonia formation. When used together, they may support growth performance more reliably than when used individually. Consistent with this, a feedlot study in finishing steers reported that TE (0.08% of dietary DM) improved G:F only when fed in combination with monensin (Felizari et al. 2025).

In our meta-analytical approach, TE supplementation occasionally improved ADG when used alone; however, the magnitude of the response was generally modest. More pronounced and consistent improvements were observed under specific nutritional conditions, such as low dietary protein intake or co-administration with monensin. These findings indicate that TE may function more effectively as a context-dependent additive rather than a universal growth promoter, potentially maximizing its benefits under particular dietary scenarios. Instead of applying TE uniformly across feeding programs, producers should consider adjusting its inclusion level based on the production system’s specific nutritional and management context. Enhanced nitrogen utilization is a key mechanism through which TE may contribute to environmental sustainability (Waghorn 2008).

### Limitations and future implications

This study has several limitations that should be considered when interpreting the findings. First, there was substantial variability in the composition and source of TE across studies, and the ratio of CT to HT was often inconsistently reported or omitted entirely. Consequently, our quantitative modeling relies on a harmonized TE metric, which may mask fundamental differences in the ruminal behavior and biological functions of CT versus HT. Therefore, our analysis necessarily treats TE at the commercial blend level and does not allow inference about CT- versus HT-specific effects or additive versus synergistic mechanisms. The assumption of synergistic or additive effects between these tannin types remains context-dependent and requires further investigations. Second, few studies employed factorial designs that would allow for robust evaluation of interactions between TE and other dietary components; therefore, interactions with different additives other than monensin (e.g., antibiotics, urea/NPN, implants, yeast/DFM) could not be systematically evaluated. This meta-analysis focused exclusively on growth performance and did not address environmental outcomes, such as nitrogen excretion or enteric methane emissions. Future research should aim to improve the chemical characterization and reporting of TE products, particularly concerning CT and HT content. Well-designed factorial experiments are needed to understand better how TE interacts with key dietary variables, including protein concentration, forage-to-concentrate ratio, and ionophores such as monensin. A critical area for further investigation is how TE supplementation affects nitrogen retention, greenhouse gas emissions, and animal performance under different nutritional contexts. Addressing these gaps will be essential to fully evaluate the role of TE in sustainable ruminant production systems.

## Conclusion

This meta-analysis integrated data from a broad range of studies to evaluate the association between TE supplementation and growth performance in growing and finishing beef cattle. Across the available literature, TE supplementation was associated with increased ADG in a nonlinear, dose-dependent pattern. Model-based analyses indicated that predicted ADG peaked at approximately 0.30% of dietary DM; however, positive responses were also observed at inclusion levels as low as 0.08% of dietary DM in some study subsets. TE supplementation was associated with improved G:F alongside changes in DMI, indicating that observed performance responses were not solely explained by intake. Effect sizes of TE inclusion were context-dependent, with larger responses observed in subsets characterized by lower protein intake and/or the presence of monensin, suggesting that TE might enhance N efficiency and(or) alter ruminal fermentation. Overall, these results indicate that TE responses are context-dependent rather than uniform. Further research is needed to refine dosing strategies and to evaluate outcomes not directly addressed in the current dataset (e.g., nitrogen excretion and methane emissions).

## Supplementary Material

skag082_Supplementary_Data

## Abbreviations

ADG: average daily gain
AIC: Akaike information criterion
CI: confidence interval
CPI: crude protein intake
CT: condensed tannin
DM: dry matter
DMI: dry matter intake
G:F: gain:feed
HT: hydrolyzable tannin
REML: restricted maximum likelihood
SE: standard error
SMD: standardized mean difference
TE: tannin extract
