# Supplementary material for: The impact of supplementing quebracho and chestnut tannin extracts on the growth performance in growing and finishing beef cattle: a meta-analytical assessment
Source: J Anim Sci. 2026 May 11;104:skag082. doi: 10.1093/jas/skag082 (PMC13188999; doi:10.1093/jas/skag082)
Supplement: skag082_Supplementary_Data [file skag082_supplementary_data.docx]

**SUPLEMENTARY MATERIAL**

**The impact of supplementing quebracho and chestnut tannin extracts on the growth performance in growing and finishing beef cattle: A meta-analytical assessment**

Mingyung Lee and Luis O. Tedeschi^*^

*Department of Animal Science, Texas A&M University, TX*

^*^Corresponding author: [luis.tedeschi@tamu.edu](mailto:luis.tedeschi@tamu.edu)

**Supplementary Figure 1.** Boxplot by gender for tannin extract (% of dietary DM).


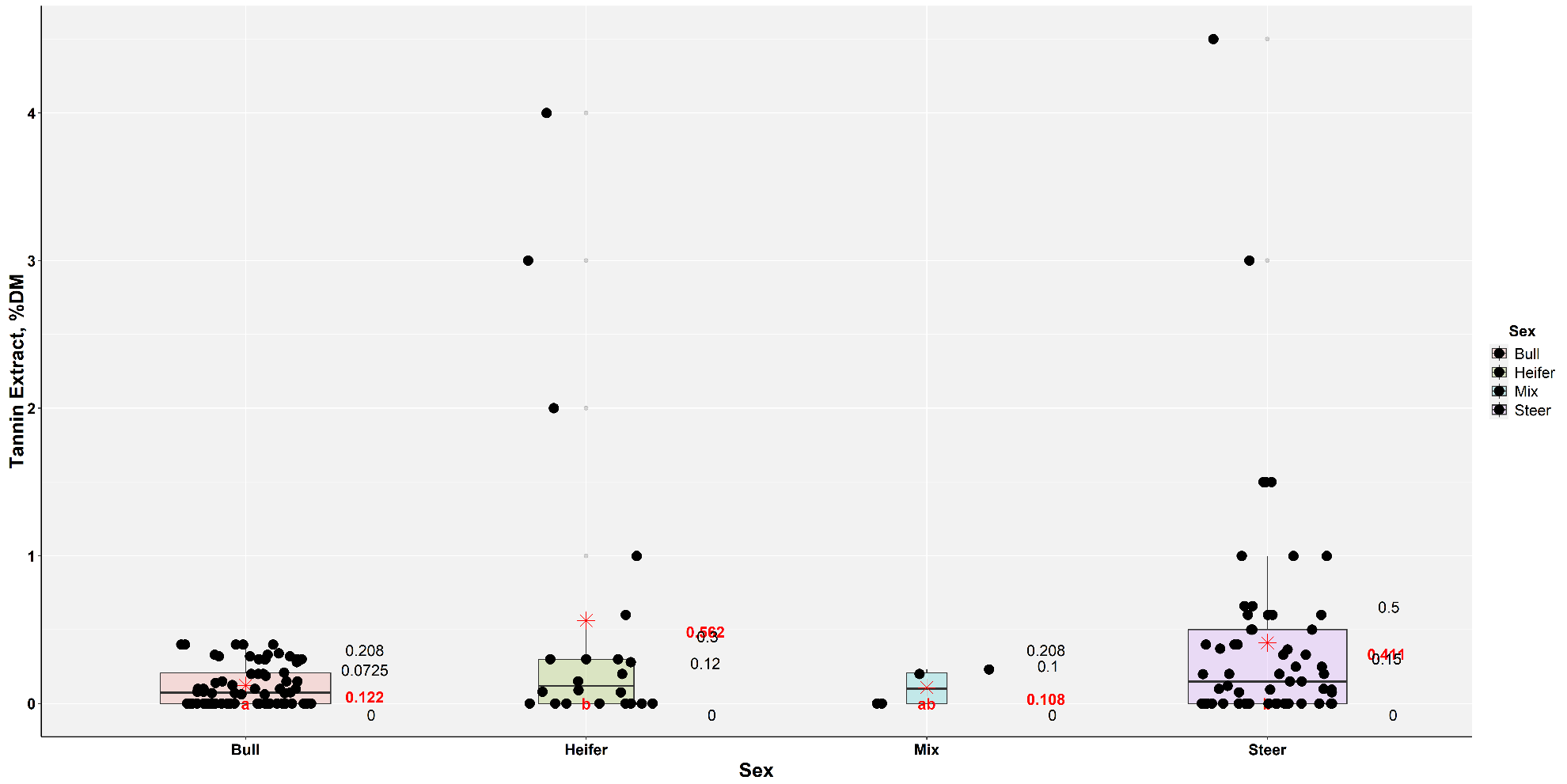


**Supplementary Figure 2.** Boxplot by gender for average daily gain (kg/d).


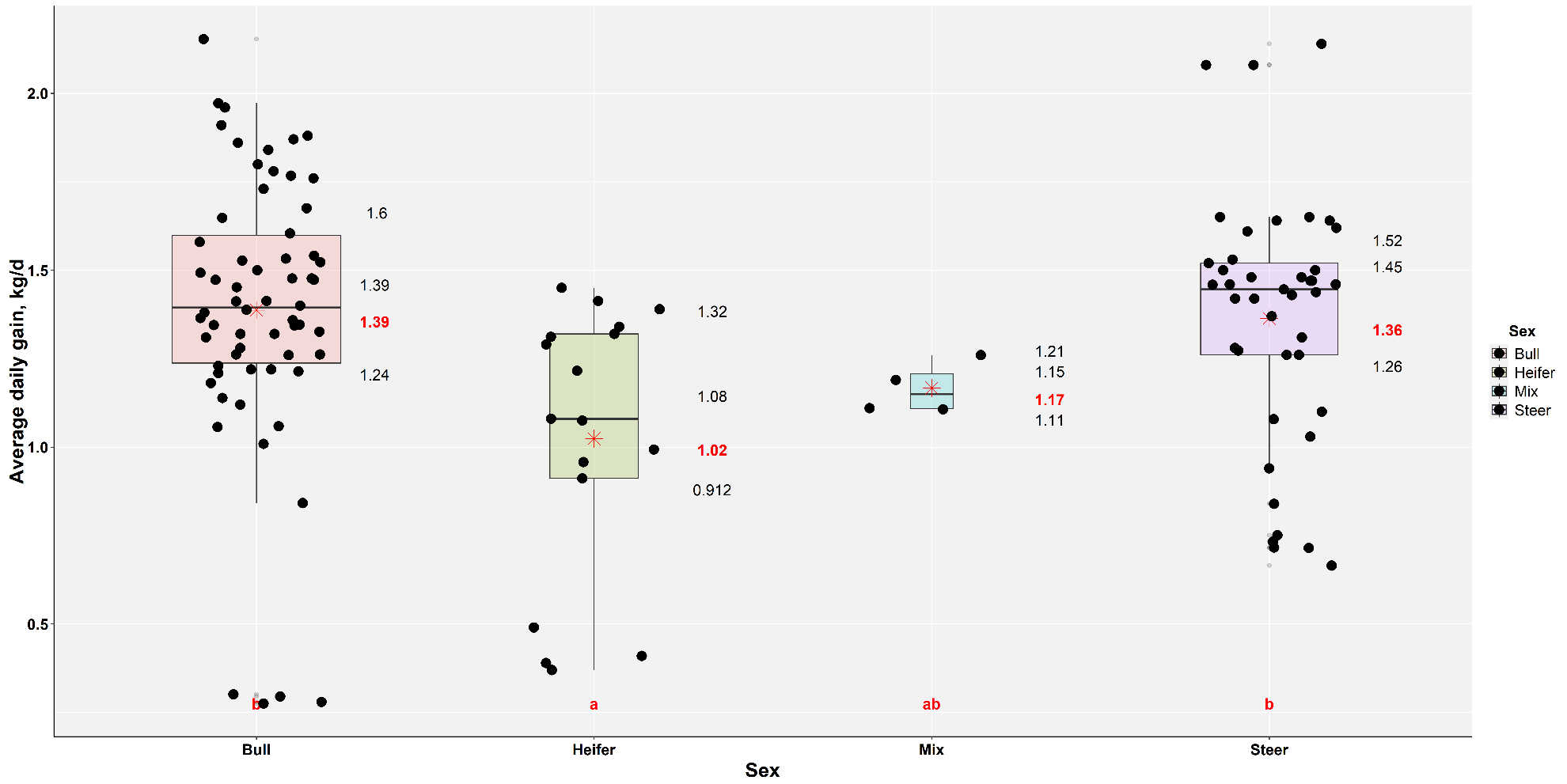


**Supplementary Figure 3.** Boxplot by gender for dry matter intake (kg/d).


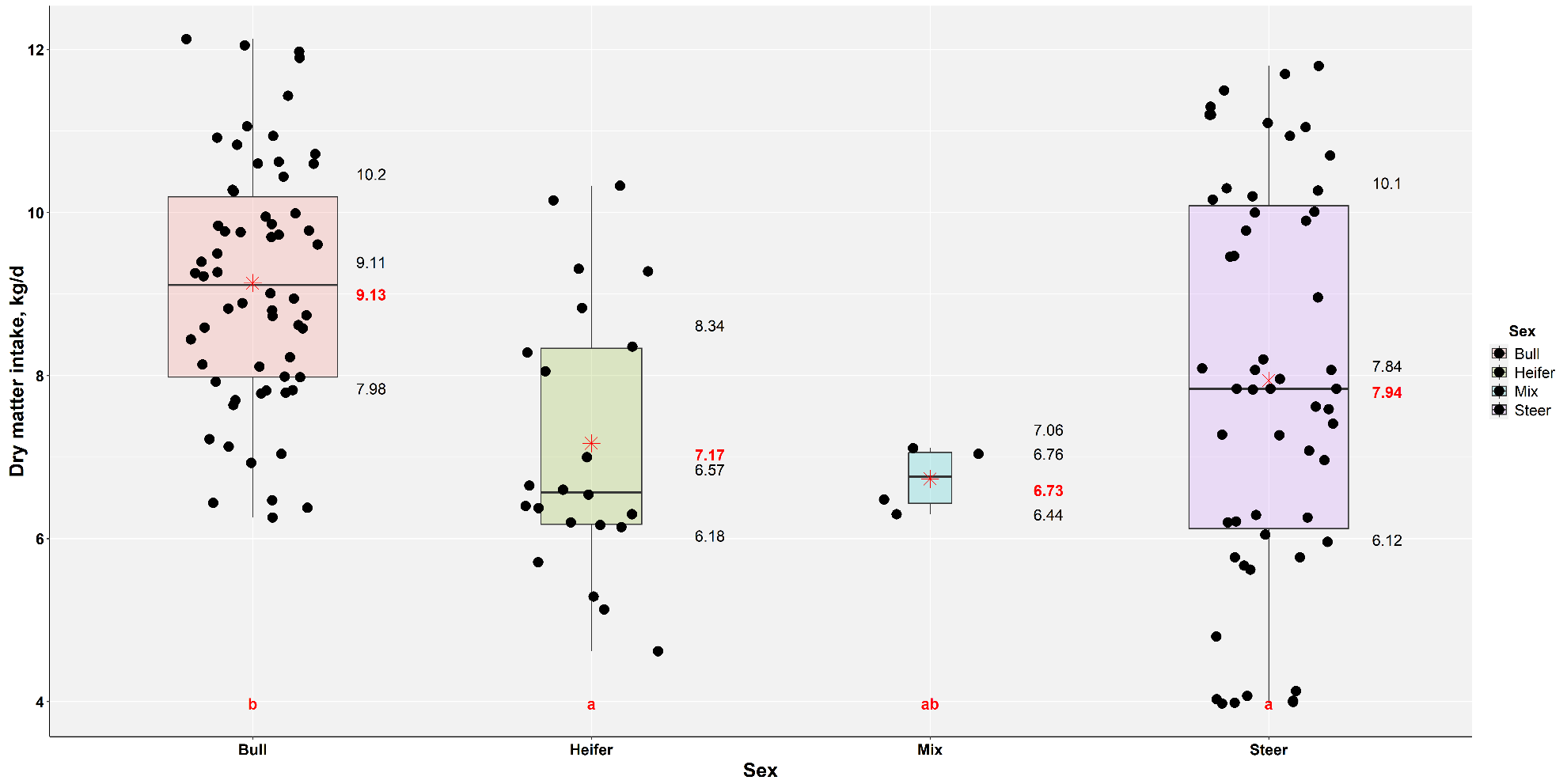


**Supplementary Figure 4.** Boxplot by gender for gain:feed (g/kg).


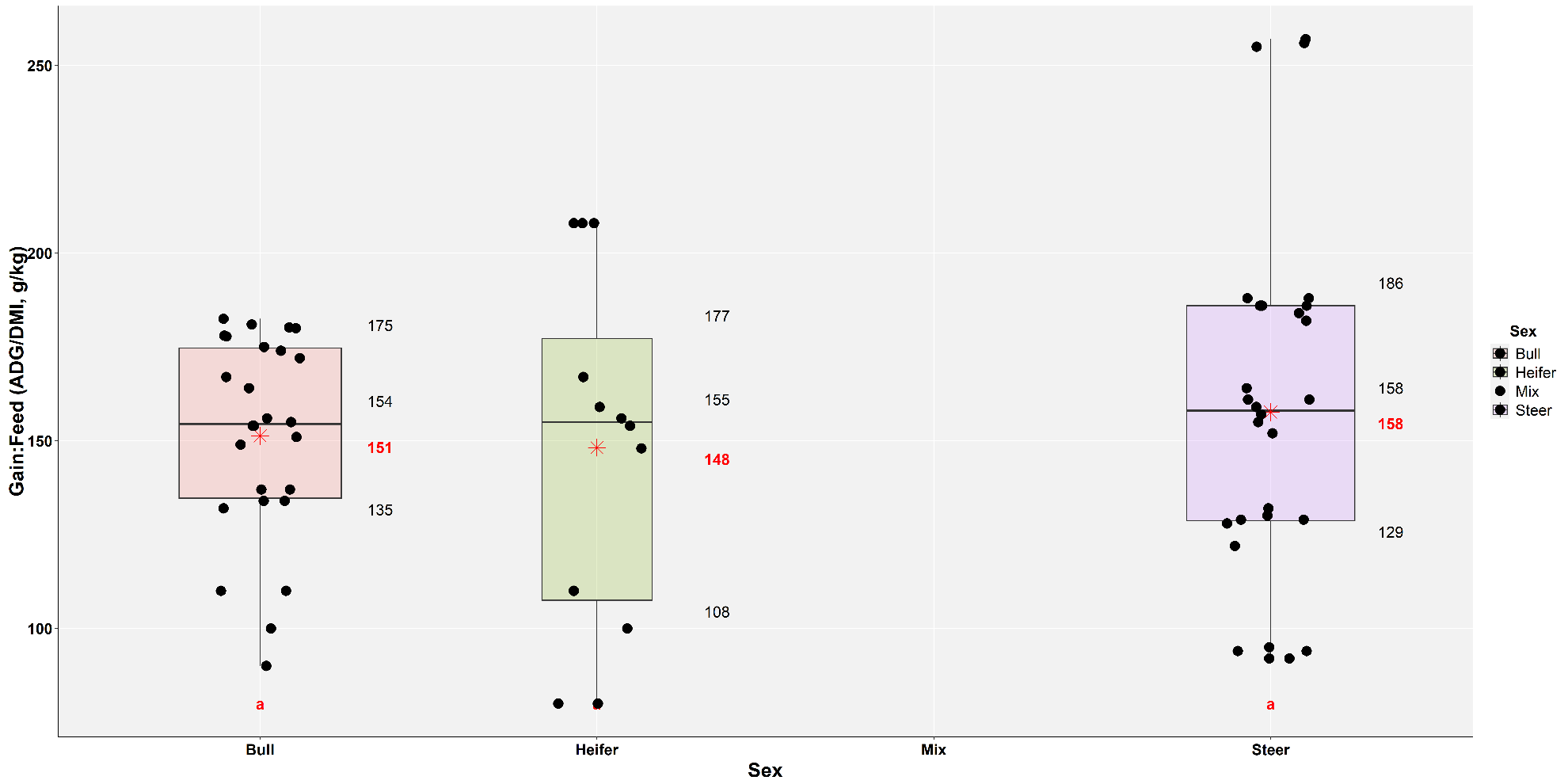


**Supplementary Figure 5.** Forest plot and meta-regression for average daily gain (ADG, kg/d) versus tannin extract (% DM). PubID refers to the publication identification number: 1 = Barajas et al. (2011b), 2 = Barajas et al. (2010), 3 = Barajas et al. (2011a), 7 = Barajas et al. (2012a), 10 = Montoya et al. (2013), 12 = Barajas et al. (2015), 13 = Mezzomo et al. (2016), 14 = Cabral et al. (2016), 15 = Rivera-Méndez et al. (2017), 16 = Ebert et al. (2017), 17 = Barrios (2017); thesis, 19 = Tabke et al. (2017), 20 = Aboagye et al. (2018), 22 = dos Anjos (2019); thesis, 25 = Demarco et al. (2021), 26 = Ferracini et al. (2024), 27 = Cidrini et al. (2022), 28 = Montano et al. (2022), 29 = Bowman et al. (2023), 30 = He et al. (2023), 31 = Magnani et al. (2023), 33 = Nascimento et al. (2024), 36 = Schilling-Hazlett et al. (2024), 39 = Manella et al. (2024b), 40 = Manella et al. (2024a), 41 = Cabral et al. (2022); internal report, 42 = Manella and Desrues (2023); internal report, and 43 = Lacherre et al. (2024). TE indicates tannin extract, and Dose represents the dose of tannin extract. Others denotes other dietary supplements, including MN (monensin), Cr (chromium), TMP (TMP Protein Enhancer^®^), TY (tylosin), VM (virginiamycin), YT (yeast), DFM (direct-fed microbes), FM (flavomycin), and Vit E (vitamin E). N indicates the number of animals, and SEM is the standard error of the mean.


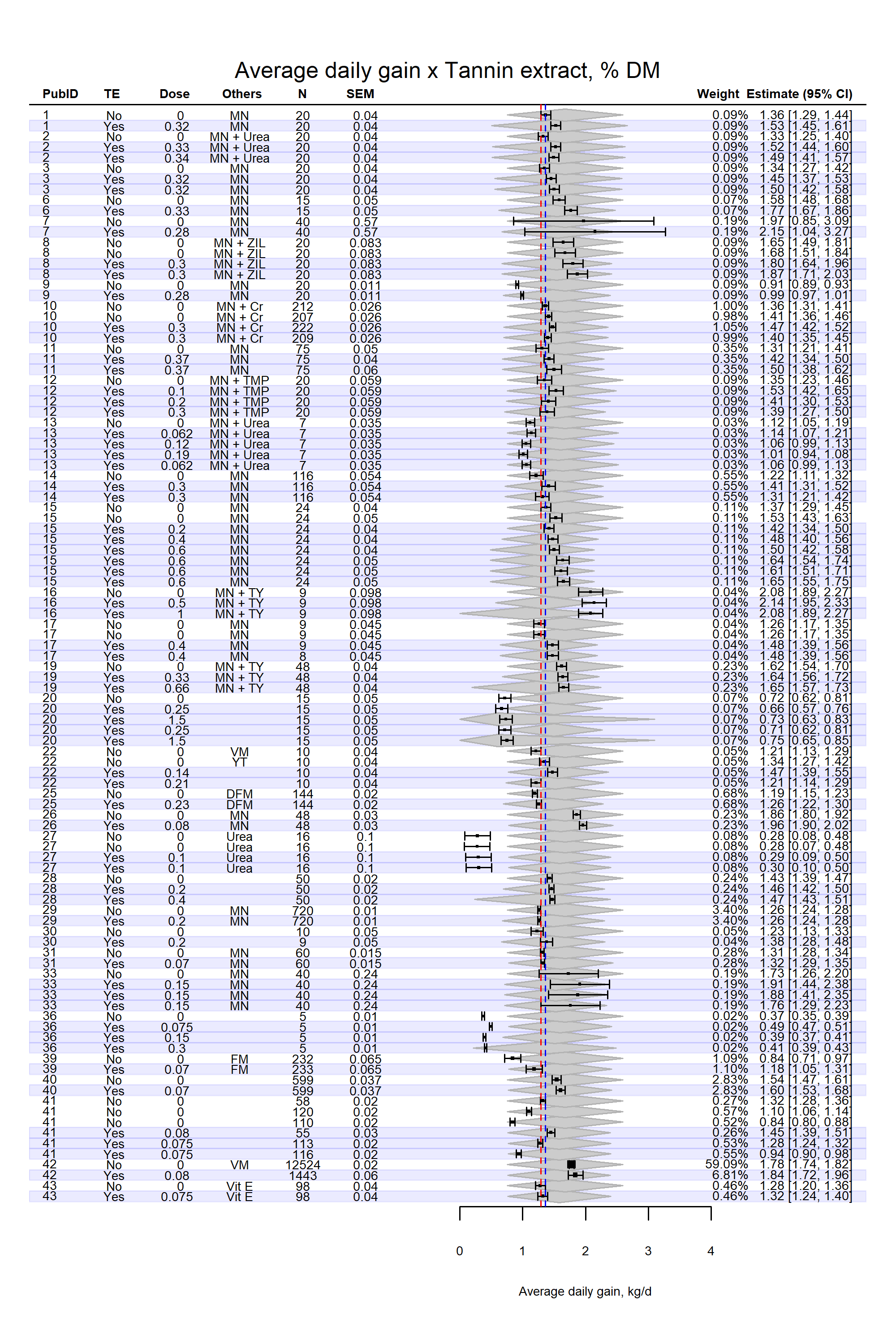


**Supplementary Figure 6.** Forest plot and meta-regression for dry matter intake (DMI, kg/d) versus tannin extract (% DM). PubID refers to the publication identification number: 1 = Barajas et al. (2011b), 2 = Barajas et al. (2010), 3 = Barajas et al. (2011a), 4 = Mezzomo et al. (2011), 6 = Barajas et al. (2012b), 7 = Barajas et al. (2012a), 8 = Barajas et al. (2013), 9 = Cervantes et al. (2013), 10 = Montoya et al. (2013), 11 = Pasinato et al. (2012); internal report, 12 = Barajas et al. (2015), 13 = Mezzomo et al. (2016), 14 = Cabral et al. (2016), 15 = Rivera-Méndez et al. (2017), 16 = Ebert et al. (2017), 18 = Piñeiro-Vázquez et al. (2018), 19 = Tabke et al. (2017), 20 = Aboagye et al. (2018), 23 = Martello et al. (2020), 24 = Norris et al. (2020), 25 = Demarco et al. (2021), 26 = Ferracini et al. (2024), 27 = Cidrini et al. (2022), 28 = Montano et al. (2022), 29 = Bowman et al. (2023), 31 = Magnani et al. (2023), 33 = Nascimento et al. (2024), 36 = Schilling-Hazlett et al. (2024), 40 = Manella et al. (2024a), 42 = Manella and Desrues (2023); internal report, and 43 = Lacherre et al. (2024). TE indicates tannin extract, and Dose represents the dose of tannin extract. Others denotes other dietary supplements, including MN (monensin), ZIL (zilpaterol hydrochloride), Cr (chromium), TMP (TMP Protein Enhancer^®^), TY (tylosin), DFM (direct-fed microbes), VM (virginiamycin), and Vit E (vitamin E). N indicates the number of animals, and SEM is the standard error of the mean.


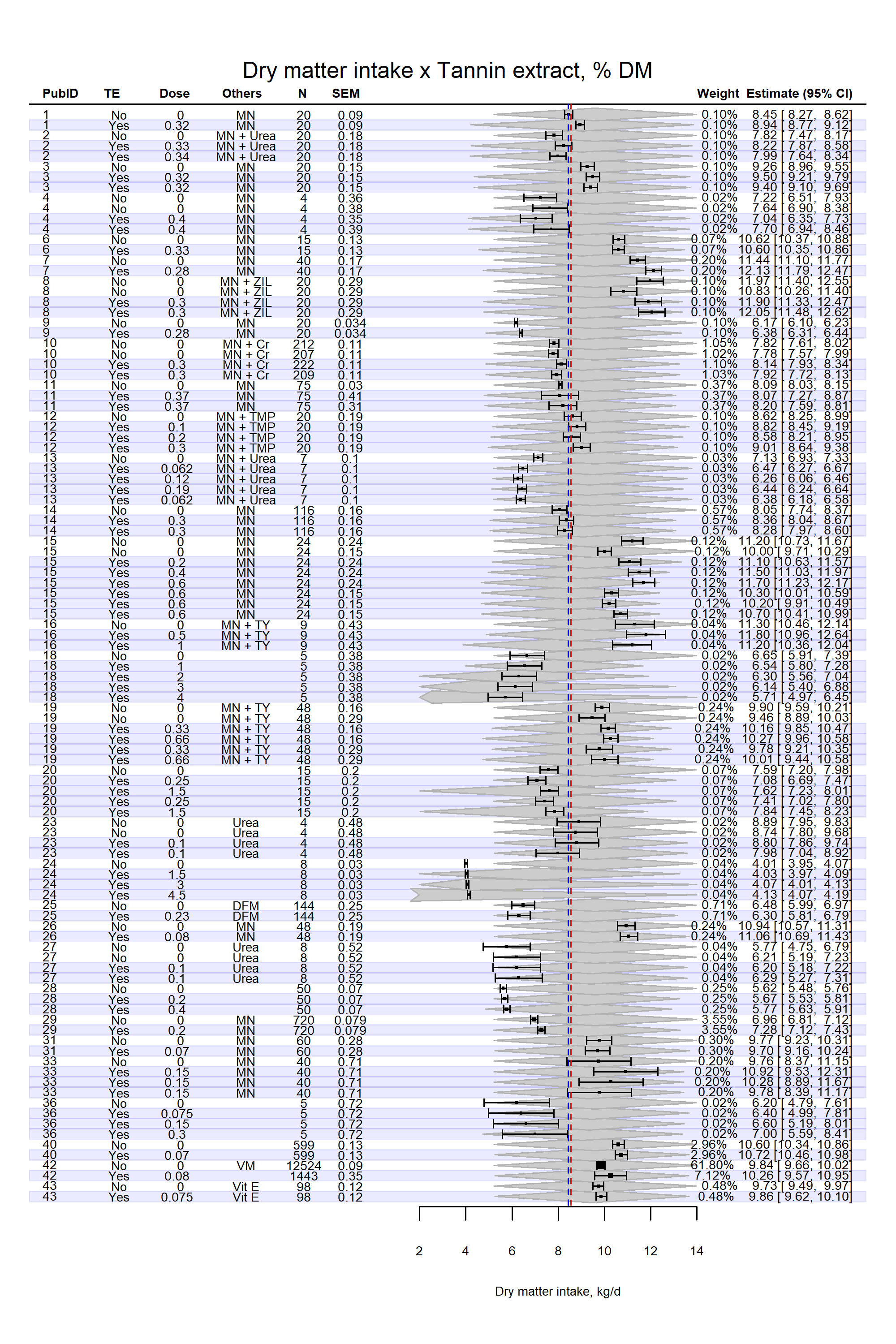


**Supplementary Figure 7.** Forest plot and meta-regression for gain:feed (G:F, g/kg) versus tannin extract (% DM). PubID refers to the publication identification number: 7 = Barajas et al. (2012a), 9 = Cervantes et al. (2013), 12 = Barajas et al. (2015), 14 = Cabral et al. (2016), 15 = Rivera-Méndez et al. (2017), 16 = Ebert et al. (2017), 19 = Tabke et al. (2017), 20 = Aboagye et al. (2018), 22 = dos Anjos (2019); thesis, 28 = Montano et al. (2022), 29 = Bowman et al. (2023), 31 = Magnani et al. (2023), 36 = Schilling-Hazlett et al. (2024), 42 = Manella and Desrues (2023); internal report, and 43 = Lacherre et al. (2024). TE indicates tannin extract, and Dose represents the dose of tannin extract. Others denotes other dietary supplements, including MN (monensin), TMP (TMP Protein Enhancer^®^), TY (tylosin), VM (virginiamycin), YT (yeast), and Vit E (vitamin E). N indicates the number of animals, and SEM is the standard error of the mean.


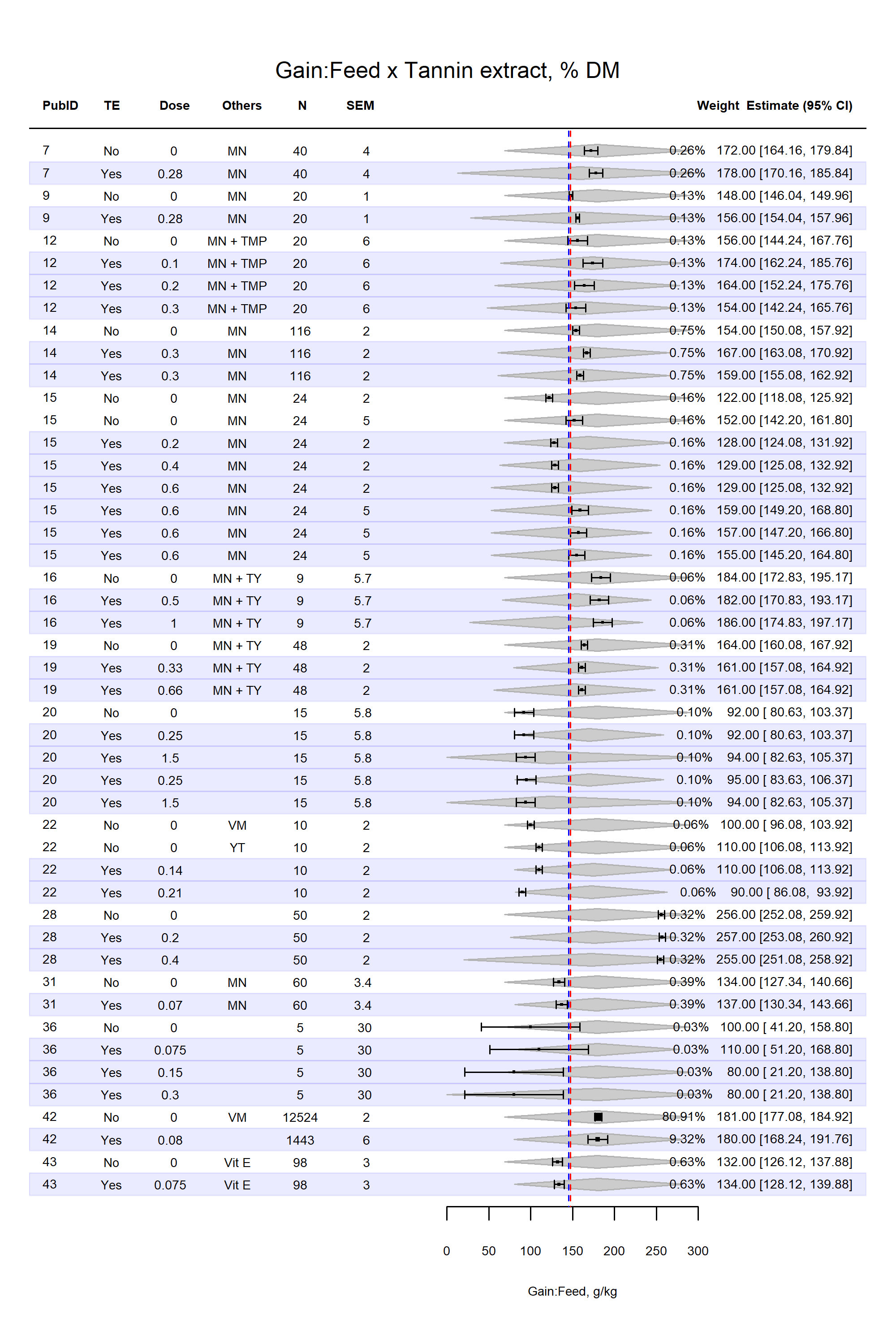


**References**

Aboagye, I. A., M. Oba, A. R. Castillo, K. M. Koenig, A. D. Iwaasa, and K. A. Beauchemin. 2018. Effects of hydrolyzable tannin with or without condensed tannin on methane emissions, nitrogen use, and performance of beef cattle fed a high-forage diet. J. Anim. Sci. 96(12):5276-5286. doi: <https://doi.org/10.1093/jas/sky352>

Barajas, R., B. J. Cervantes, S. C. Arechiga, M. A. Espino, L. R. Flores, A. Camacho, and J. A. Romo. 2011a. W296 Effect of length feeding additional tannins-extract on feedlot-performance of finishing-bulls. J. Anim. Sci. 89(Suppl. 1):615. (Abstr.)

Barajas, R., B. J. Cervantes, A. Camacho, E. A. Velazquez, M. A. Espino, F. Juarez, L. R. Flores, and M. Verdugo. 2010. W384 Condensed tannins supplementation on feedlot performance of growing bulls. J. Anim. Sci. 88(Suppl. 2):711. (Abstr.)

Barajas, R., B. J. Cervantes, A. Camacho, M. Verdugo, M. A. Espino, L. R. Flores, J. A. Romo, E. A. Velazquez, and J. J. Lomeli. 2011b. W294 Influence of addition of tannins-extract in low concentration of dietary dry matter on feedlot-performance of bulls. J. Anim. Sci. 89(Suppl. 1):615. (Abstr.)

Barajas, R., B. J. Cervantes, M. A. Espino, A. Camacho, M. Verdugo, L. R. Flores, S. C. Aréchiga, J. J. Lomeli, and J. A. Romo. 2012a. T343 Influence of tannins extract addition on feedlot-performance of bulls fed sorghum-based diets. J. Anim. Sci. 90(Suppl. 3):372-373. (Abstr.)

Barajas, R., B. J. Cervantes, M. A. Espino, A. Camacho, M. Verdugo, L. R. Flores, J. J. Lomeli, and J. A. Romo. 2012b. W284 Effect of tannins extract supplementation on feedlot performance and plasma urea nitrogen of yearling bulls fed dryground corn-based diets containing corn-DDG and cane molasses. J. Anim. Sci. 90(Suppl. 3):599-600. (Abstr.)

Barajas, R., B. J. Cervantes, M. A. Espino, A. Camacho, M. Verdugo, L. R. Flores, and J. A. Romo. 2013. T11 Interaction of tannin extract and zilpaterol hydrochloride supplementation on feedlot performance of bulls. J. Anim. Sci. 91(Suppl. 2):8. (Abstr.)

Barajas, R., B. J. Cervantes, J. A. Vázquez, A. Camacho, and J. A. Romo. 2015. M327 Influence of a tannins extract preparation supplementation level on the feedlot performance of feedlot bullocks. J. Anim. Sci. 93(Suppl. 3):123. (Abstr.)

Barrios, M. M. 2017. Evaluation of the inclusion of tannins in the diet of feedlot-finished steers: productive efficiency, Universidad Nacional de Nordeste, Corrientes, Argentina. Thesis. (In Spanish)

Bowman, S. M., L. K. Fuerniss, J. D. Cameron, J. L. Beckett, N. Panciroli, M. Manella, C. Guthrie, and B. J. Johnson. 2023. Replacement of monensin with a tannin blend in calf-fed Holstein steer diets. Plains Nutrition Council Spring Conference p.93. (Abstr.)

Cabral, C., J. Baeck, M. Manella, and O. Desrues. 2022. The use of Silvafeed BX as natural alternative to monensin in commercial feedlots in Argentina. Silvateam S.p.A. Internal Report BX608:1-3. Argentina.

Cabral, C., A. Lopez Da Silva, J. J. Couderc, D. Colombatto, and R. Barajas. 2016. 1573 Influence of tannins extract and monensin supplementation on performance of feedlot heifers in Argentina. J. Anim. Sci. 94(Suppl. 5):764-764. (Abstr.) doi: <https://doi.org/10.2527/jam2016-1573>

Cervantes, B. J., A. Camacho, J. A. Vazquez, M. A. Espino, T. J. Heras, L. R. Flores, J. J. Lomeli, and R. Barajas. 2013. T8 Influence of tannins extract supplementation on feedlot performance and plasma urea nitrogen of nonimplanted growing heifers. J. Anim. Sci. 91(Suppl. 2):7. (Abstr.)

Cidrini, I. A., Y. T. Granja-Salcedo, L. F. Prados, L. T. Kishi, G. R. Siqueira, and F. D. Resende. 2022. Effect of tannin extract associated with two levels of non-protein nitrogen in the supplement on performance, ruminal parameters, and microbial diversity of grazing Nellore cattle during the growing phase at dry season. Anim. Feed Sci. Technol. 286:115269. doi: <https://doi.org/10.1016/j.anifeedsci.2022.115269>

Demarco, C. F., S. Paisley, R. Goodall, C. C. Brauner, and S. Lake. 2021. Effects of bacterial DFM and tannins on measures of immunity and growth performance of newly weaned beef calves. Livest. Sci. 250:104571. doi: <https://doi.org/10.1016/j.livsci.2021.104571>

dos Anjos, E. 2019. Additives in intensive finishing of beef cattle on pasture during the dry season, Universidade Federal de Mato Grosso, Mato Grosso, Brazil. Thesis. (In Portuguese)

Ebert, P. J., E. A. Bailey, A. L. Shreck, J. S. Jennings, and N. A. Cole. 2017. Effect of condensed tannin extract supplementation on growth performance, nitrogen balance, gas emissions, and energetic losses of beef steers. J. Anim. Sci. 95(3):1345-1355. doi: <https://doi.org/10.2527/jas.2016.0341>

Ferracini, J. G., A. L. J. Lelis, D. Polli, M. B. Gasparim, L. T. Feba, I. N. d. Prado, and D. D. Millen. 2024. Feedlot performance of Nellore bulls fed high-concentrate diets containing the association of tannins and saponins with sodium monensin. Rev. Bras. Zootec. 53:e20230104. doi: <https://doi.org/10.37496/rbz5320230104>

He, T., G. Yi, J. Li, Z. Wu, Y. Guo, F. Sun, J. Liu, C. Tang, S. Long, and Z. Chen. 2023. Dietary supplementation of tannic acid promotes performance of beef cattle via alleviating liver lipid peroxidation and improving glucose metabolism and rumen fermentation. Antioxidants 12(9)doi: <https://doi.org/10.3390/antiox12091774>

Lacherre, A., J. M. Bollatti, L. Soave, G. Enriquez, R. Irurtia, C. Cabral, and N. Di Lorenzo. 2024. Use of blends of tannins and saponins in feedlot finishing diets: effects on productive performance. 47th Argentine Congress of Animal Production (Suppl. 1):258. (Abstr.)

Magnani, E., T. H. Silva, L. Sakamoto, M. Q. Manella, F. M. G. N. Dias, M. E. Mercadante, D. Henry, J. O. S. Marcatto, E. M. Paula, and R. H. Branco. 2023. Tannin-based product in feedlot diet as a strategy to reduce enteric methane emissions of Nellore cattle finished under tropical conditions. Transl. Anim. Sci. 7(1):txad048. doi: <https://doi.org/10.1093/tas/txad048>

Manella, M., and O. Desrues. 2023. The effects of Silvafeed BX as natural alternative to virginiamycin in a commercial feedlot. Silvateam S.p.A. Internal Report BX609:1-2. Argentina.

Manella, M. Q., A. Campanini, C. Boin, E. Paula, R. B. Arnandes, and V. Lopes. 2024a. PSXII-24 Use of a commercial blend of tannins as feed additive for feed lot cattle on large pen trial improves carcass weight and carcass feed conversion. J. Anim. Sci. 102(Suppl. 3):778-779. (Abstr.) doi: <https://doi.org/10.1093/jas/skae234.877>

Manella, M. Q., R. Coan, W. Mesquita, E. Paula, and R. B. Arnandes. 2024b. PSXI-20 Proteic dry supplements enriched with a blend of tannin-based extract enhance the performance of grass-fed cattle during the rainy season in tropical regions. J. Anim. Sci. 102(Suppl. 3):762-763. (Abstr.) doi: <https://doi.org/10.1093/jas/skae234.860>

Martello, H. F., N. F. De Paula, R. W. Teobaldo, J. T. Zervoudakis, M. A. Fonseca, L. S. Cabral, J. K. L. Rocha, A. T. Mundim, and E. H. B. K. Moraes. 2020. Interaction between tannin and urea on nitrogen utilization by beef cattle grazing during the dry season. Livest. Sci. 234:103988. doi: <https://doi.org/10.1016/j.livsci.2020.103988>

Mezzomo, R., P. V. R. Paulino, M. M. Barbosa, T. da Silva Martins, M. F. Paulino, K. S. Alves, D. I. Gomes, and J. P. I. dos Santos Monnerat. 2016. Performance and carcass characteristics of young cattle fed with soybean meal treated with tannins. Anim. Sci. J. 87(6):775-782. doi: <https://doi.org/10.1111/asj.12486>

Mezzomo, R., P. V. R. Paulino, E. Detmann, S. C. Valadares Filho, M. F. Paulino, J. P. I. S. Monnerat, M. S. Duarte, L. H. P. Silva, and L. S. Moura. 2011. Influence of condensed tannin on intake, digestibility, and efficiency of protein utilization in beef steers fed high concentrate diet. Livest. Sci. 141(1):1-11. doi: <https://doi.org/10.1016/j.livsci.2011.04.004>

Montano, M. F., P. H. V. Carvalho, J. O. Chirino-Romero, B. C. Latack, J. Salinas-Chavira, and R. A. Zinn. 2022. Influence of supplemental condensed tannins on initial 112-d feedlot growth-performance and characteristics of digestion of calf-fed Holstein steers. Transl. Anim. Sci. 6(1):txac024. doi: <https://doi.org/10.1093/tas/txac024>

Montoya, A., J. J. Bermudez, and R. Barajas. 2013. T10 Influence of tannins extract and organic chromium supplementation on feedlot performance. J. Anim. Sci. 91(Suppl. 2):7. (Abstr.)

Nascimento, K. S., L. E. L. M. Bomfim, V. R. M. Couto, M. B. Silva, A. L. A. Lopes, M. H. M. R. Fernandes, M. Q. Manella, M. V. C. F. Junior, and J. J. R. Fernandes. 2024. Growth performance and carcass characteristics of bulls fed tannins associated or not with monensin. Transl. Anim. Sci.:Submitted. doi: <https://doi.org/10.1093/tas/txae136>

Norris, A. B., W. L. Crossland, L. O. Tedeschi, J. L. Foster, J. P. Muir, W. E. Pinchak, and M. A. Fonseca. 2020. Inclusion of quebracho tannin extract in a high-roughage cattle diet alters digestibility, nitrogen balance, and energy partitioning. J. Anim. Sci. 98(3):skaa047. doi: <https://doi.org/10.1093/jas/skaa047>

Pasinato, A., G. Morao, V. Pianetti, J. Adrien, M. Medina, R. Aramburu, and C. Cabral. 2012. Evaluation of tannin inclusion in feedlot-finished animals: effects on animal response. Silvateam S.p.A. Internal Report:1-6. Uruguay.

Piñeiro-Vázquez, A. T., G. Jiménez-Ferrer, J. A. Alayon-Gamboa, A. J. Chay-Canul, A. J. Ayala-Burgos, C. F. Aguilar-Pérez, and J. C. Ku-Vera. 2018. Effects of quebracho tannin extract on intake, digestibility, rumen fermentation, and methane production in crossbred heifers fed low-quality tropical grass. Trop. Anim. Health Prod. 50(1):29-36. doi: <https://doi.org/10.1007/s11250-017-1396-3>

Rivera-Méndez, C., A. Plascencia, N. Torrentera, and R. A. Zinn. 2017. Effect of level and source of supplemental tannin on growth performance of steers during the late finishing phase. J. Appl. Anim. Res. 45(1):199-203. doi: <https://doi.org/10.1080/09712119.2016.1141776>

Schilling-Hazlett, A., E. J. Raynor, L. Thompson, J. Velez, S. Place, and K. Stackhouse-Lawson. 2024. On-farm methane mitigation and animal health assessment of a commercially available tannin supplement in organic dairy heifers. Animals 14(1)doi: <https://doi.org/10.3390/ani14010009>

Tabke, M. C., J. O. Sarturi, M. L. Galyean, S. J. Trojan, J. C. Brooks, B. J. Johnson, J. Martin, J. Baggerman, and A. J. Thompson. 2017. Effects of tannic acid on growth performance, carcass characteristics, digestibility, nitrogen volatilization, and meat lipid oxidation of steers fed steam-flaked corn–based finishing diets. J. Anim. Sci. 95(11):5124-5136. doi: <https://doi.org/10.2527/jas2017.1464>
